# Desiderata for normative models of synaptic plasticity

**Published:** 2023-08-09

**Authors:** Colin Bredenberg, Cristina Savin

**Affiliations:** 1 Center for Neural Science, New York University, New York, NY 10003, USA; 2 Mila-Quebec AI Institute, 6666 Rue Saint-Urbain, Montréal, QC H2S 3H1; 3 Center for Data Science, New York University, New York, NY 10011, USA

**Keywords:** computational neuroscience, learning, synaptic plasticity

## Abstract

Normative models of synaptic plasticity use a combination of mathematics and computational simulations to arrive at predictions of behavioral and network-level adaptive phenomena. In recent years, there has been an explosion of theoretical work on these models, but experimental confirmation is relatively limited. In this review, we organize work on normative plasticity models in terms of a set of desiderata which, when satisfied, are designed to guarantee that a model has a clear link between plasticity and adaptive behavior, consistency with known biological evidence about neural plasticity, and specific testable predictions. We then discuss how new models have begun to improve on these criteria and suggest avenues for further development. As prototypes, we provide detailed analyses of two specific models – REINFORCE and the Wake-Sleep algorithm. We provide a conceptual guide to help develop neural learning theories that are precise, powerful, and experimentally testable.

## 1. Introduction

Our identities change with time, gradually reshaping our experiences. We remember, we associate, we learn. However, we are only beginning to understand how changes in our minds arise from underlying changes in our brains. Of the many features of neural architecture that are altered over time, from the biophysical properties of individual neurons to the creating or pruning of synapses between neurons, changes in the strength of existing synapses have long been among the most prominent candidates for the neural substrate of longitudinal perceptual and behavioral change, because many synaptic connections are easily modified, and these modifications can persist for extended periods of time (Bliss and Collingridge, 1993). Further, synaptic modification has been associated with many of the brain’s critical adaptive functions, including memory (Martin et al., 2000), experience-based sensory development (Levelt and Hübener, 2012), operant conditioning (Ohl and Scheich, 2005; Fritz et al., 2003), and compensation for stroke (Murphy and Corbett, 2009) or neurodegeneration (Zigmond et al., 1990). However, beyond these associations, a precise link between plasticity and adaptive behaviors of interest is currently lacking.

Here, we distinguish ‘normative’ modeling approaches from other alternatives, demonstrate why they show promise for establishing this link, and outline a set of desiderata which articulate how recent progress on normative plasticity models strengthens the link between plasticity and system-wide adaptive phenomena. To provide concrete examples of these principles in action, in Appendices C and D we provide worked tutorials on two complementary canonical normative plasticity models—REINFORCE (Williams, 1992) for reinforcement learning, and the Wake-Sleep algorithm for unsupervised learning (Dayan et al., 1995; Hinton et al., 1995)—and illustrate their successes and failures to match our desiderata.

### 1.1. Phenomenological, mechanistic, and normative plasticity models

**Figure 1:**
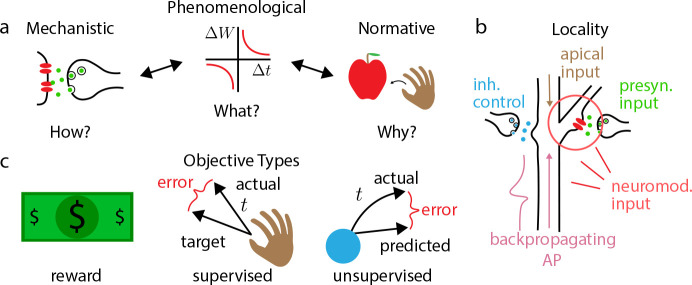
Defining normative modeling. **a.** Spectrum of synaptic plasticity models. Mechanistic models show how detailed biophysical interactions produce observed plasticity, phenomenological models concisely describe what changes in experimental variables (e.g. post-pre relative spike timing Δt) affect plasticity (ΔW), and normative models explain why the observed plasticity implements capabilities that are useful to the organism. **b.** Schematic illustrating the range of local variables that may be available for synaptic plasticity. These include, but are not limited to: backpropagating action potentials from the soma, apical dendritic input, pre and postsynaptic activity, neuromodulatory signals, and potentially inhibitory input from local microcircuitry. **c.** Classes of objective function used in normative plasticity theories. Reward-based objectives involve only feedback about how well the organism or network performed, whereas supervised objectives provide explicit targets for network output. By contrast, unsupervised objectives do not require any form of explicit feedback to train the network.

We distinguish between three partially overlapping types of model: phenomenological, mechanistic, and normative (Fig. 1a) (Levenstein et al., 2020). The focus of this review is normative plasticity models, but to understand their importance, we first describe their relationship to their counterparts.

In the simplest terms, a phenomenological model’s focus is on describing experimental data: the primary goal is to concisely summarize relationships between observed variables. As an example, many early studies of spike-timing-dependent plasticity (STDP) described the relationship between plasticity and the relative timing of pre- and post-synaptic spikes with exponential curves fit to data (Zhang et al., 1998; Dan and Poo, 2004; Sjöström et al., 2010). Such models can reduce the complexity of data, providing interpretability and, to some extent, predictive power. They are incomplete descriptions of the biophysical processes that form the causal link between spike times and plasticity, but extract and summarize important features of the data on which subsequent theories and models can build.

A mechanistic model attempts to explain a set of experimental results in terms of causal interactions between biophysical quantities. For instance, since the initial characterization of STDP, a plethora of studies have emerged characterizing in detail the interactions between backpropagating action potentials (Magee and Johnston, 1997), dendritic morphological properties (Froemke et al., 2005; Letzkus et al., 2006; Sjöström and Häusser, 2006), local membrane voltage, NMDA ion channel properties, and calcium-sensitive molecules near the synapse. Mechanistic models (Graupner and Brunel, 2010) characterize how these variables all collectively contribute to the strengthening or weakening of the synapse. As a consequence of their depth and breadth, mechanistic models can often provide predictions that are outside of the scope of the original experiment, and provide useful targets for experimental manipulation.

The distinction between phenomenological models and mechanistic models is not always completely crisp, especially in areas where our scientific understanding is progressing rapidly. In nascent mechanistic models, there often exist ‘black boxes’ that specify interactions between known biophysical quantities, without a precise understanding of whether or how these interactions are implemented (Craver, 2007). Because they lack a direct relation to well-understood biophysics, these ‘black boxes’ act in essentially the same way as variables do in a phenomenological model. In this way, we can see that there exists a spectrum between phenomenological and mechanistic models, and that oftentimes, mechanistic models grow from phenomenological ones. However, there is more to the spectrum: while phenomenological and mechanistic models articulate how synaptic plasticity works, they do not explain *why* it exists in the brain, i.e. what its importance is for neural circuits, behavior, or perception. To answer this question with any precision requires an appeal to normative modeling.

Normative models aim answer this ‘why’ question by connecting plasticity to observed network-level or behavioral-level phenomena, including memory formation (Hopfield, 1982) and consolidation (Benna and Fusi, 2016; Clopath et al., 2008; Fusi et al., 2005), reinforcement learning (Frémaux and Gerstner, 2016), and representation learning (Hinton et al., 1995; Oja, 1982; Rao and Ballard, 1999; Savin et al., 2010). This class of plasticity model, in our view, employs a fundamentally different set of methodologies from phenomenological or mechanistic models, in order to provide the missing link between plasticity and function. Guided by the intuition that plasticity processes have developed on an evolutionary timescale to near-optimally perform adaptive functions, normative plasticity theories are typically ‘top-down’, in that they begin with a set of prescriptions about how synapses ‘should’ modify in order to optimally perform a given learning-based function. Subsequently, with varying degrees of success, these theories attempt to show that real biology matches or approximates this optimal solution. As an example, an increasing body of literature is establishing a correspondence between classical reinforcement learning algorithms (Williams, 1992) and reward-modulated Hebbian synaptic plasticity models of learning in the brain (Frémaux and Gerstner, 2016). This process is ongoing, and though experimental support for such forms of plasticity are growing (Gerstner et al., 2018), much work remains to be done. Similar efforts are underway to construct approximations to the backpropagation algorithm which can serve as models of neural plasticity (Marschall et al., 2020; Lillicrap et al., 2020; Richards and Lillicrap, 2019; Urbanczik and Senn, 2014). Here, we will review classical normative plasticity approaches and discuss recent efforts to improve upon them.

## 2. Desiderata for normative models

One of the biggest challenges for a normative model of synaptic plasticity is its connection to biology: artificial neural networks with simulated synapses (synaptic weight parameters) that adapt to improve performance on any of a variety of functions from sensory processing (LeCun et al., 1989; Krizhevsky et al., 2012), to motor learning (Heess et al., 2017; Hafner et al., 2019), to abstract game learning (Silver et al., 2017; Vinyals et al., 2019) are much more accessible to mathematical and empirical investigation than the neural circuits implementing these functions in the brain. Compared to the simulations and mathematical analysis used to explore machine learning algorithms, neuroscience experiments are time-consuming and expensive. Further, network simulations provide total access to neural activations, stimuli, and synaptic parameters over the whole course of learning, whereas any one neuroscience experiment can only reveal a very small amount about what is going on in a circuit. Therefore, it is a major challenge to identify how to improve normative models with relatively limited access to experimental data confirming or rejecting their predictions.

In what follows, we will articulate a set of desiderata that can serve as both an organizing tool for understanding the contributions of recent normative plasticity modeling efforts and as intermediate objectives for the development of new models in the absence of explicit experimental rejection or confirmation of older work. We will argue that each principle is desirable for some combination of the following reasons: first, it may help ensure that the plasticity model actually qualifies as normative; second, it may require a model to accommodate known facts about biology; third, it may ensure that models can be compared properly to existing experimental literature and generate genuinely testable experimental predictions. Most of these desiderata are relatively intuitive and simple. However, it has proven incredibly difficult for existing models of any adaptive cognitive phenomenon—from sensory representation learning, to associative memory formation, to reinforcement learning—to satisfy all desiderata in tandem.

### 2.1. Improving performance

One way to view the normative approach is that it attempts to organize the diversity of synaptic dynamics existing within a neural system into the simplest explanatory framework possible for what functions the system’s plasticity subserves. Usually, this framework is mathematical for pragmatic reasons: mathematics provides the precision and power necessary to establish clear relationships between plasticity and function. In particular, viewing neural plasticity as an approximate optimization process has been very fruitful (Lillicrap et al., 2020; Richards et al., 2019), wherein synaptic modifications progressively reduce a scalar loss function. This process can be divided into two steps: articulating an appropriate objective, and subsequently demonstrating that a synaptic plasticity mechanism improves performance on that objective.

It can be extremely difficult to reduce the full range of functions a given circuit must perform to a scalar objective function, but as we will show subsequently, the conceptual benefits can be immense. On one side, picking too simple an objective function runs the risk of ignoring many functions a system is required to perform. For instance, early normative theories of learning in sensory systems show how synaptic plasticity could minimize the objective function underlying principal component analysis (PCA) (Oja, 1982), but merely representing the principal components of an incoming sensory stream is an inadequate characterization of sensory processing for several reasons. PCA can capture only second-order properties (mean and covariance) of naturalistic stimuli and does not perform the highly nonlinear processing required for cortical neurons to exhibit gain control capabilities (Simoncelli and Heeger, 1998) and texture (Ziemba et al., 2016) and object class (Rust and DiCarlo, 2010) selective responses. A given synaptic plasticity mechanism may only be able to minimize a restricted subset of objectives, and for a normative theory, the set of possible objectives that can be minimized must encompass a wide range of functions that the brain is known to subserve. Beyond principal component analysis, many modern models of unsupervised representation learning use objectives for training hierarchical generative models (e.g. the evidence lower bound (ELBO) which underlies the Wake-Sleep algorithm (Dayan et al., 1995) and predictive coding (Rao and Ballard, 1999), and allows for multilayer, nonlinear representation learning). On the other side, selecting too flexible an objective function can run the risk of ‘overfitting’ experimental data, a problem that is particularly salient for Bayes-optimal accounts of neuroscientific and psychological phenomena (Bowers and Davis, 2012). As an extreme example, if we were to postulate that the ‘objective’ of a neural system is to behave exactly as it is observed to behave experimentally, i.e. everything in a neural system happens precisely as was ‘intended’, then the normative project becomes vacuous: the model provides neither conceptual simplification nor predictive power beyond what was observed experimentally, and has consequently failed to provide a useful explanation of the data. Therefore, the quality of an objective function is determined by both how many phenomena it is able to explain and how simple it is.

Normative theories of synaptic plasticity developed to date usually involve some combination of supervised, unsupervised, or reinforcement learning objectives (Fig. 1c). The choice of objective function for a neural system is laden with philosophical assumptions about the system’s functional utility, and can exert a huge influence on the resultant form and scope of applicability of the synaptic plasticity model. For instance, supervised learning usually involves the existence of either an internal or external teacher. If the teacher is external, such a learning mechanism could only be leveraged under the very specific and comparatively rare conditions in which the organism is being overtly taught, as is the case, for instance, in some models of zebra finch song learning (Fiete et al., 2007). If the teacher is internal, a plausible normative theory is limited in the types of knowledge the ‘self-supervisor’ may reasonably construct and provide (for instance, motor error signals (Gao et al., 2012; Bouvier et al., 2018) or saccade information indicating that a visual scene has changed (Illing et al., 2021)). Generative modeling is a form of unsupervised learning that postulates that a sensory system is actively building a probabilistic model of its sensory inputs, which can be used to simulate possible future outcomes and perform Bayesian reasoning (Fiser et al., 2010). This vision of sensory coding is popular both for its ability to accommodate normative plasticity theories (Rao and Ballard, 1999; Dayan et al., 1995; Kappel et al., 2014; Bredenberg et al., 2021) and for its philosophical vision of sensory processing as a form of advanced model building, beyond simple sensory transformations. However, model construction is only indirectly useful for many tasks involving rewards and planning, and so such plasticity would have to occur concomitantly with reward-based (Frémaux and Gerstner, 2016) or motor (Gao et al., 2012; Feulner and Clopath, 2021) learning. Furthermore, alternative perspectives on sensory processing exist, including those based on maximizing the information about a sensory stimulus contained in a neural population (Attneave, 1954; Atick and Redlich, 1990) subject to metabolic efficiency constraints (Tishby et al., 2000; Simoncelli and Olshausen, 2001), and those based on ‘contrastive methods’ (Oord et al., 2018; Illing et al., 2021), where a self-supervising internal teacher encourages the neural representation of some stimuli to grow closer together, while encouraging others to grow more discriminable.

Evaluating which objective function (or functions) best explains the properties of a neural system is very hard: while some forms of objective function may have discriminable effects on plasticity (e.g. supervised vs. unsupervised learning (Nayebi et al., 2020)), others are even provably impossible to distinguish. As a simple example, suppose that we have an $N^{r}$ dimensional single-layer neural network receiving $N^{s}$ dimensional stimuli through an $N^{r}\times N^{s}$ dimensional weight matrix W. We have the response given by:

(1) $$r=f\left(Ws\right),$$

where f(⋅) is a tanh nonlinearity. Now suppose that some setting of synaptic weights $W^{*}$ minimizes an objective function ℒ, i.e. $ℒ(W^{*})\leq ℒ\left(W\right)\;\forall W$. We might be tempted to argue that because $W^{*}$ minimizes ℒ,ℒ must be the objective that the system is minimizing. However, there are an infinite variety of alternative objectives that share this same minimum (Appendix A). This motivates the idea that for a given dataset, it is very plausible that one objective $\left(\tilde{ℒ}\right)$ can *masquerade* as another (ℒ). In some cases, complex objective functions can masquerade as simple objectives, which may only be epiphenomenal. For instance, it has been hypothesized that synaptic modifications may preserve the balance between inhibitory and excitatory inputs to a cell (Vogels et al., 2011); recent theories have proposed that this E/I balance may only be a consequence of a more advanced theory of sensory predictive coding (Brendel et al., 2020). In other cases, philosophically distinct frameworks, such as generative modeling, information maximization, or denoising may simply produce similar synaptic plasticity modifications because the frameworks often overlap heavily (Vincent et al., 2010), and may not be distinguishable on simple datasets without targeted experimental attempts to disambiguate between the two perspectives.

Furthermore, not every function performed by biological systems has been adequately incorporated into a simple optimization framework. For example, though the Hebbian plasticity rule used in Hopfield networks endows model circuits with associative memory, the utility of learning is characterized by the dynamical attractor structure it embeds in the neural circuit, rather than by its direct minimization of an objective function (Hopfield, 1982). In addition, the notion that some parts of the brain may have synaptic plasticity mechanisms for representation learning while other parts have plasticity for reinforcement learning suggests that the brain may be better viewed as a collection of interacting systems with only partially overlapping goals. This multiagent (Zhang et al., 2021) formulation of learning has intuitive appeal, because it can decompose broad objectives like survival into a series of intermediate objectives carried out by individual systems. Such a formulation could help explain how locality emerges, i.e. why synapses do not need information about distant neural circuits in order to improve performance. However, with this additional appeal comes additional conceptual and mathematical complexity, because improving performance on one objective could very easily harm the performance of other systems. Therefore, insofar as a collection of neural circuits and plasticity mechanisms *can* be viewed as acting in concert to improve a unified objective, simple optimization is the preferable perspective.

Having addressed many difficulties associated with choosing a good objective function, we now move to difficulties involved in demonstrating that a particular synaptic plasticity rule decreases a chosen objective^1^. How could such a property be proven? For a particular plasticity rule to reduce an objective, we need to show that the following principle holds:

(2) $$\begin{array}{c} ℒ(W+\Delta W)<ℒ(W)\\ \Rightarrow ℒ(W+\Delta W)-ℒ(W)<0, \end{array}$$

for some update ΔW determined by the plasticity rule. If we accept the additional supposition that ΔW is very small, we can employ the first order Taylor approximation (treating W as a flattened vector of length $N^{r}\times N^{s}):\;ℒ(W+\Delta W)\approx ℒ(W)+\frac{dℒ}{dW}(W{)}^{T}\Delta W$. Substituting this approximation into our reduction criterion, we have after cancellation:

(3) $$\frac{dℒ}{dW}(W{)}^{T}\Delta W<0.$$

This shows that for small weight updates (slow learning rates), the inner product between a synaptic learning rule ΔW and the gradient of the selected loss function ℒ(W) with respect to the weight change must be negative. The simplest way to ensure that this is true is for ΔW to equal a small scalar λ times the negative gradient of the loss $\left(-\lambda \frac{dℒ}{dW}(W{)}^{T}\frac{dℒ}{dW}(W)=-\lambda {∥\frac{dℒ}{dW}(W)∥}_{2}^{2}<0\right)$. If this were true, plasticity would be guaranteed to improve performance on the objective ℒ. Unfortunately, for even the simplest neural networks and objective functions, naive methods of calculating this gradient will prove to be nonlocal (see Appendix B for a simple example). Thus, the critical challenge for normative theories of synaptic plasticity is finding ways that neural networks can find synaptic modifications ΔW that demonstrably have a negative inner product with the gradient of a desired objective ℒ, while still allowing the neural network to satisfy biologically realistic locality constraints. However, it is important to note that if an update ΔW reduces any one objective function, then it also reduces an infinite number of similar alternative objective functions (Appendix A); therefore it is perhaps best to think of normative plasticity models in terms of the family of objective functions that they minimize—committing to any one particular objective within that family reflects the predilections of the theorist, not the system.

Different normative studies demonstrate that Eq. 3 holds by different methods. Some studies show empirically across many simulations that this inner product is negative (Lillicrap et al., 2016; Marschall et al., 2020). However, these demonstrations alone do not answer the following questions: how would we know that the network would still perform well if a different task were chosen, or if the network’s architecture were different, or if various elements of the simulated plasticity mechanism were changed? A simulation has relatively limited power to extrapolate beyond its immediate results, especially when the neuron models used in large-scale network simulations are often very reductive (Gerstner and Kistler, 2002) and when small changes in simulated network parameters can effect large qualitative differences in network behavior (Xiao et al., 2021). Further, a battery of *in silico* simulations under a variety of different parameter settings and circumstances rapidly begins to suffer the curse of dimensionality, becoming almost as extensive as the collection of *in vivo* or *in vitro* experiments that it is attempting to explain. As such, simulation-based justifications suffer from a lack of conciseness and an inability to easily address counterfactuals.

For this reason, much focus in the field has been devoted to constructing mathematical arguments as to why Eq. 3 should hold for a given local synaptic plasticity rule. Some plasticity rules amount to stochastic approximations to the true gradient (Williams and Zipser, 1989; Scellier and Bengio, 2017) and some are systematically biased but maintain a negative inner product under reasonable assumptions (Bredenberg et al., 2021; Dayan et al., 1995; Amari and Nakahara, 1999; Meulemans et al., 2020). Mathematical analysis allows one to know quite clearly when a particular plasticity rule will decrease a loss function, and identifies how plasticity mechanisms should change with changes in the network architecture or environment. However, analysis is often only possible under restrictive circumstances, and it is often necessary to supplement mathematical results with empirical simulations in order to demonstrate that the results extend to more general, more realistic circumstances.

### 2.2. Locality

Biological synapses can only change strengths using chemical and electrical signals available at the synapse itself. ‘Locality’ refers to the idea that a postulated synaptic plasticity mechanism should only refer to variables that could be conceivably available at a given synapse (Fig. 1b). Though locality may seem like an obvious requirement for any theory of biological function, for synaptic plasticity it presents a great mystery: how does a system as a whole, whose success or failure is determined by the joint action of many neurons distributed across the entire brain, communicate information to individual synapses about how to improve? The success of most machine learning algorithms relies on nonlocal, even global, propagation of learning signals, including backpropagation (Werbos, 1974; Rumelhart et al., 1985) (See Appendix B), backpropagation through time (Werbos, 1990), and real-time recurrent learning (Williams and Zipser, 1989).

Despite its importance as a guiding principle for normative theories of synaptic plasticity, locality is a slippery concept, primarily because of our insufficient understanding of the precise battery of biochemical signals available to a synapse, and how those signals could be used to approximate quantities required by theories. As a simple example, many normative theories require information about the pre- and postsynaptic firing rates of a neuron, similar to Hebb’s Postulate (Hebb, 1949). However, neurons predominately communicate to one another through discrete action potentials, and additional cellular machinery would be required to form an estimate of pre- and postsynaptical firing rates based on backpropagating action potentials from the soma and on postsynaptic potentials. Whether a plasticity rule derived from normative principles involves rate or spike-based information is often a function of the neuron model used in the theory, and it is often difficult to formulate predictions about how a realistic, non-idealized neuron should exactly modify its synapses based on over-simplified models. Therefore, normative theories typically declare success when some standard of plausibility is reached, where derived plasticity rules roughly match the experimental literature (Payeur et al., 2021) or only require reasonably simple functions of postsynaptic and pre-synaptic activity that a synapse could hypothetically approximate (Oja, 1982; Scellier and Bengio, 2017; Williams, 1992).

In normative models of synaptic plasticity, the requirement of locality is in perpetual tension with the general requirement for some form of ‘credit assignment’ (Lillicrap et al., 2020; Richards et al., 2019), i.e. a mechanism capable of signaling to a neuron that it is ‘responsible’ for a network-wide error, and should modify its synapses to reduce errors. Depending on a network’s objective, a system’s credit assignment mechanism *could* take a wide variety of forms, some small number of which may only require information about the pre- and post-synaptic activity of a cell (Oja, 1982; Pehlevan et al., 2015, 2017; Obeid et al., 2019; Brendel et al., 2020), but many of which appear to require the existence of some form of error (Scellier and Bengio, 2017; Lillicrap et al., 2016; Akrout et al., 2019) or reward-based (Williams, 1992; Fiete et al., 2007; Legenstein et al., 2010) signal.

The extent to which a credit assignment signal postulated by a normative theory meets the standards of ‘locality’ depends heavily on the nature of the signal. For instance, there is growing support for the idea that neuromodulatory systems, distributing dopamine (Otani et al., 2003; Calabresi et al., 2007; Reynolds and Wickens, 2002), norepinephrine (Martins and Froemke, 2015), oxytocin (Marlin et al., 2015), and acetylcholine (Froemke et al., 2013; Guo et al., 2019; Hangya et al., 2015; Rasmusson, 2000; Shinoe et al., 2005) signals can propagate information about reward (Guo et al., 2019), expectation of reward (Schultz et al., 1997), and salience (Hangya et al., 2015) diffusely throughout the brain to induce or modify synaptic plasticity in their targeted circuits. Therefore, it may be reasonable for normative theories to postulate that synapses have access to global reward or reward-like signals, without violating the requirement that plasticity be affected only by locally-available information (Frémaux and Gerstner, 2016).

Locality as a desideratum serves as a heuristic stand-in for the requirement that a normative model must be eventually held to the standard of experimental evidence. This is not to say that normative models cannot postulate neural mechanisms that have not yet been observed experimentally. However, for such an exercise to be constructive, the theory should clearly articulate how it deviates from the current state of the experimental field, and how these deviations can be tested (Section 2.7; see Appendices C and D for concrete examples of this process). Furthermore, the process of mathematical abstraction necessitates approximation (Cartwright and McMullin, 1984): constraining a normative theory to adhere to ‘locality’ without necessarily requiring a perfect correspondence to experimental data allows normative theories to strive to capture the essence of synaptic learning processes without becoming mired in technical details.

### 2.3. Architectural plausibility

The learning algorithm implemented by a plasticity model often requires specific architectural motifs to exist in a neural circuit in order to deliver reward, error, or prediction signals. These might include diffuse neuromodulatory projections (Fig. S1b) or neuron-specific top-down synapses onto apical dendrites (Fig. S2c). Such architectural features (or alternative, isomorphic motifs) are *required* for the learning algorithm in question, and are known to exist in a wide range of cortical areas. However, normative plasticity models should not depend on circuit features that have been demonstrated not to exist in the modeled system, because spurious architectural features can be used to ‘cheat’ at achieving locality by postulating unrealistic credit assignment mechanisms (see Appendix B). Further, models lacking important features of neural circuits can be difficult to relate to experimental data. In what follows, we will highlight several particularly important architectural motifs that have been the focus of recent work.

**Figure 2:**
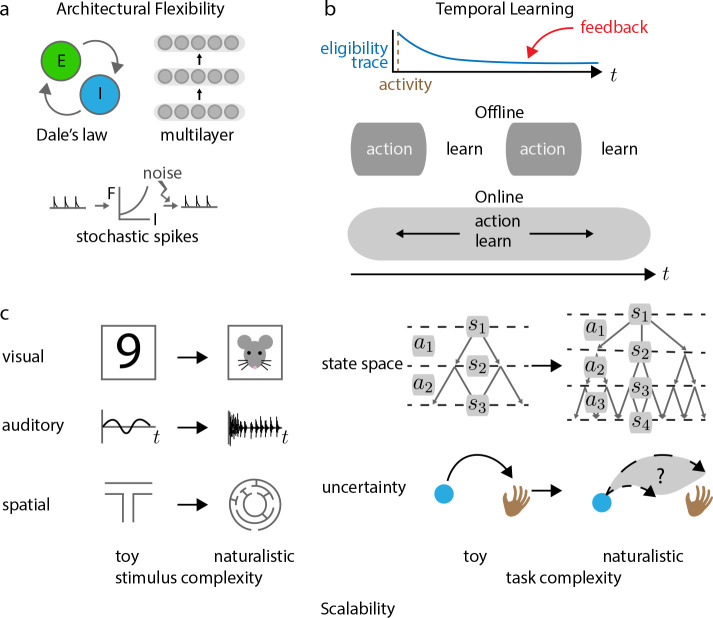
Architecture and scalability considerations for normative plasticity models. **a.** Features of realistic biological networks that normative plasticity theories should be able to account for: separation of excitatory and inhibitory neuron populations; stochastic and spiking input-output functions for individual neurons; and multilayer, recurrent connectivity. **b.** For actions in the past to be associated with delayed supervisory or reinforcement signals, plasticity algorithms require a mechanism of temporal association. One candidate is the ‘eligibility trace,’ which stores information about coactivity throughout time locally to a synapse, and subsequently modifies synaptic connections when paired with feedback information. Learning can occur offline, where some or all synaptic modification occurs in the absence of action or perception by the organism. Alternatively, it can occur online, where the organism acts and learns simultaneously. **c.** Stimuli (left) and task structure (right) can become complex in many ways. Different sensory features (e.g. visual, auditory, or spatial information) can all be made more naturalistic by training networks on stimuli organisms are exposed to and learn from in natural environments. Further, tasks can be made more naturalistic by increasing the number of action options (*a*) and sequential state (*s*) transitions required for a network to achieve its goals and by adding uncertainty into the task.

Contrary to the highly reduced deterministic rate-based models typically used in machine learning, neurons communicate through roughly discrete action potentials. Further, they exhibit numerous forms of variability due in part to synaptic failures and constant receipt of task-irrelevant signals (Fig. 2a) (Faisal et al., 2008). Normative theories which employ rate-based activations (Bredenberg et al., 2020; Scellier and Bengio, 2017) or which assume that the input-output function of neurons is approximately linear (Oja, 1982), may not extend to the more realistic discrete, stochastic, and highly nonlinear setting. Further, by ignoring spike timing, such theories inherently produce plasticity rules that ignore the precise relationship between pre- and post-synaptic spike times, and will consequently be unable to capture STDP results. This both limits the expressive power of such models, and prevents their experimental validation. Fortunately, several methods which were originally formulated using rate-based models have subsequently been extended to spiking network models to great effect. Reward-based Hebbian plasticity based on the REINFORCE algorithm (Appendix C) (Williams, 1992) has been generalized to stochastic spiking networks (Frémaux et al., 2013), while backpropagation approximations (Murray, 2019) and predictive coding methods (Rao and Ballard, 1999) have subsequently extended to deterministic spiking networks (Bellec et al., 2020; Brendel et al., 2020). Therefore, a lack of a generalization to spiking networks is not necessarily a death knell for a normative theory, but many existing theories lack either an explicit generalization to spiking or a clear relationship to STDP, and the mathematical formalism that defines these methods may require significant modification to accommodate the change.

Real biological networks have a diversity of cell types with different neurotransmitters and connectivity motifs. At the bare minimum, a normative model must be able to accommodate Dale’s Law (Fig. 2a), which stipulates that the neurotransmitters released by a neuron are either excitatory or inhibitory, but not both (for the most part (O’Donohue et al., 1985)). Though this might seem like a simple principle, enforcing Dale’s principle can seriously damage the performance of artificial neural networks without careful architectural considerations (Cornford et al., 2021). Furthermore, the mathematical results of *many* canonical models of synaptic modification rely on symmetric connectivity between neurons, including Hopfield networks (Hopfield, 1982), Boltzmann machines (Ackley et al., 1985), contrastive Hebbian learning (Xie and Seung, 2003), and predictive coding (Rao and Ballard, 1999); this symmetry is partially related to the symmetric connectivity required by the backpropagation algorithm (Appendix B). Symmetric connectivity means that the connection from neuron A to neuron B must be the same as the reciprocal connection from neuron B to neuron A. It inherently violates Dale’s Law, because it means that entirely excitatory and entirely inhibitory neurons can never be connected to one another: the positive sign for one synapse and the negative sign for the reciprocal connection violates symmetry. Some models, such as Hopfield networks (Sompolinsky and Kanter, 1986) and equilibrium propagation (Ernoult et al., 2020) have been extended to demonstrate that moderate deviations from symmetry can exist and still preserve function. Further, a recent mathematical reformulation of predictive coding has demonstrated that inter-layer symmetric connectivity is not necessary (Golkar et al., 2022). Therefore, recent results indicate that many canonical models believed to depend on symmetric connectivity can be improved upon.

Many early plasticity models, including Oja’s rule (Oja, 1982) and perceptron learning (Rosenblatt, 1958), as well as more modern model recurrent network models focused on learning temporal tasks (Murray, 2019) are designed to greedily optimize layer-wise objectives, and their mathematical justifications do not generalize to multi-layer architectures. Though greedy layer-wise optimization may be sufficient for some forms of unsupervised learning (Illing et al., 2021), a method that cannot account for how credit assignment signals are passed between cortical areas will not in general be able to support many complex supervised or reinforcement learning tasks humans are known to learn (Lillicrap et al., 2020). Generalizing layer-local learning to multi-layer objective functions has been the focus of much recent work: many multi-layer models can be seen as generalizations of perceptron learning (Bengio, 2014; Hinton et al., 1995; Rao and Ballard, 1999), with other models such as those derived from similarity matching (Pehlevan et al., 2017) receiving similar treatment (Obeid et al., 2019). We will refer to this form of multi-layer signal propagation as ‘spatial’ credit assignment, and will refer to relaying information across time as ‘temporal’ credit assignment (Fig. 2b; Section 2.4). As we will discuss in the next section, models that do not support temporal credit assignment will not be able to account for learning in inherently sequential tasks.

### 2.4. Temporal credit assignment

Because so many learned biologically-relevant tasks involving temporal decision-making (Gold and Shadlen, 2007) or working memory (Compte et al., 2000; Wong and Wang, 2006; Ganguli et al., 2008) inherently leverage information from the past to inform future behavior, and because neural signatures associated with these tasks exhibit rich recurrent dynamics (Brody et al., 2003; Shadlen and Newsome, 2001; Mante et al., 2013; Sohn et al., 2019), many aspects of learning in the brain require a normative theory of synaptic plasticity that works in recurrent neural architectures and provides an account of temporal credit assignment.

Temporal credit assignment is an important point of failure of modern deep learning methods, in part due to the inherent instabilities involved in performing gradient descent on recurrent neural architectures (Bengio et al., 1994). That models unconstrained in their correspondence to biology have difficulties handling temporal signals should be some indication of the difficulties posed by temporal credit assignment for normative theories of synaptic plasticity. However, recent improvements in neural architectures, including gated recurrent units (Chung et al., 2014) and long short-term memory units (Hochreiter and Schmidhuber, 1997), as well as sequential reinforcement learning methods (Mnih et al., 2015; Arjona-Medina et al., 2019; Hung et al., 2019; Raposo et al., 2021), have combined to produce several high-profile advances on inherently temporal, naturalistic tasks like game-playing (Silver et al., 2017) and natural language processing (Devlin et al., 2018; Radford et al., 2018). This may indicate that the time is ripe to begin incorporating new developments in deep learning into normative plasticity models.

As it currently stands, the majority of normative synaptic plasticity models focus only on spatial credit assignment, which presents distinct challenges when compared to temporal credit assignment (Marschall et al., 2020). In fact, many theories that provide a potential solution to spatial credit assignment do so by requiring networks to relax to a ‘steady-state’ on a timescale much faster than inputs (Hopfield, 1982; Scellier and Bengio, 2017; Bredenberg et al., 2020; Xie and Seung, 2003; Ackley et al., 1985), which effectively prevents networks from having the rich, slow internal dynamics required for many temporal motor (Hennequin et al., 2012) and working memory (Wong and Wang, 2006) tasks. Other methods appear to be agnostic to the temporal properties of their inputs, but have not yet been combined with existing plasticity rules that perform approximate temporal credit assignment within local microcircuits (Murray, 2019; Bellec et al., 2020).

While most normative theories focus on spatial credit assignment, some new algorithms do provide potential solutions to temporal credit assignment, through either explicit approximation of real time recurrent learning (Marschall et al., 2020; Bellec et al., 2020; Murray, 2019), by leveraging principles from control theory (Gilra and Gerstner, 2017; Alemi et al., 2018; Meulemans et al., 2022), or by leveraging principles of stochastic circuits that are fundamentally different from traditional explicit gradient-based calculation methods (Bredenberg et al., 2020; Miconi, 2017). Many use what is called an ‘eligibility trace’ (Gerstner et al., 2018) (Fig. 2b)—a local synaptic record of coactivity—to identify associations between rewards and neural activity that may have occurred much further in the past. We suggest that these models capture something fundamental about learning across time, and that much work remains to combine these with spatial learning rules to construct normative models of full spatiotemporal learning.

### 2.5. Combining learning and active performance

Similar to the importance of understanding temporal credit assignment in the brain, it is critical to understand how learning in the brain relates to continuous action and perception in an environment (Fig. 2b). The relationship between learning and active performance in the brain can vary widely depending on the experimental context: learning-related changes can occur concomitantly with action (Bittner et al., 2015; Sheffield et al., 2017; Grienberger and Magee, 2022) (‘online’ learning), during brief periods of quiescence between trials (Pavlides and Winson, 1989; Bönstrup et al., 2019; Liu et al., 2021), or over periods of extended sleep (Gulati et al., 2017; Eschenko et al., 2008; Girardeau et al., 2009) (‘offline’ learning). Therefore, whether a normative plasticity model uses offline or online learning should be determined by the experimental context, be it for instance rapid place cell reorganization in new environments, or long timescale memory consolidation.

However, many classical algorithms—especially those that support multi-layer spatial credit assignment (Ackley et al., 1985; Xie and Seung, 2003; Dayan et al., 1995)—are constrained to modeling only offline learning, because they require distinct training phases, during at least one phase of which activity of neurons is driven for *learning*, rather than performative purposes. It has not been clear whether such algorithms are fundamentally offline, or whether the space of phenomena that they can model can be expanded until recently. Some existing two-phase normative algorithms, such as the Wake-Sleep algorithm (Appendix D) (Hinton et al., 1995; Dayan et al., 1995), have be adapted such that the second phase becomes indistinguishable from perception (Bredenberg et al., 2020; Ernoult et al., 2020). Other recent models allow for simultaneous multiplexing of top-down learning signals and bottom-up inputs (Payeur et al., 2021), which enables online learning. These results suggest that future work may fruitfully adapt existing offline algorithms to provide good models of explicitly online learning in the brain.

### 2.6. Scaling in dimensionality and complexity

A point often underappreciated in computational neuroscience (and possibly over-appreciated in machine learning) is that models of learning in the brain need to be able to scale to handle the full complexity of the problems a given model organism has to solve. As obvious as this sounds, it is a point that can be difficult to verify: how can we guarantee that adding more neurons and more complexity will not make a particular collection of plasticity rules more effective? As a case study, consider REINFORCE ((Williams, 1992); Appendix C), an algorithm which, for the most part, satisfies our other desiderata for normative plasticity for the limited selection of tasks in naturalistic environments which are explicitly rewarded. However, though REINFORCE demonstrably performs better than its progenitor weight perturbation (Jabri and Flower, 1992), as the dimensionality of its stimuli, the number of neurons in the network, and the delay time between neural activity and reward increases, the performance of the algorithm decays rapidly, both analytically and in simulations (Werfel et al., 2003). This is primarily caused by high variance of gradient estimates provided by the REINFORCE algorithm, and is only partially ameliorated by existing methods that reduce its variance (Bredenberg et al., 2021; Ranganath et al., 2014; Mnih and Gregor, 2014; Miconi, 2017). Thus, adding additional complexity to the network architecture actually *impairs* learning.

We do not mean to imply that all normative plasticity algorithms should be demonstrated to meet human-level performance, or even that they should match state-of-the-art machine learning methods. Machine learning methods profit in many ways from their biological implausibility: they use stochastic backpropagation, which is demonstrably biologically implausible (Appendix B) but which benefits from very low variance gradient estimates (Werfel et al., 2003); they share weights across topographically distant space in convolutional neural networks (Fukushima and Miyake, 1982); they use rate-based units, which generally perform better than spiking units (Neftci et al., 2019); and they are usually deterministic, which obviates the need for redundancy (increased neuron numbers) and increased computational demand. Beyond machine learning methods, the human brain itself has orders of magnitude more neural units and synapses than have ever been simulated on a computer, all of which are capable of processing totally in parallel. Therefore, direct comparison to the human—or any—brain is also not fair. We propose the far softer condition that as the complexity of input stimuli and tasks increase, within the range supported by current computational power, plasticity rules derived from normative theory should continue to perform well both in simulation and, preferably, analytically. Further, the performance of normative plasticity algorithms can fruitfully be compared to existing machine learning methods as long as the comparison is performed for realistic network architectures with identical conditions, as in (Bredenberg et al., 2021; Payeur et al., 2021; Marschall et al., 2020; Bartunov et al., 2018).

Complexity is multifaceted, and involves features of both stimulus and task (Fig. 2c). Even stimuli with very high dimensional structure can fail to capture critical features of naturalistic stimuli, as evidenced by the wide gap in difficulty involved in constructing convincing models that synthesize images with low-level naturalistic features (orientation, contrast, texture (Portilla and Simoncelli, 2000)) compared to models that capture high-level image features (object identity (Rezende et al., 2014; Goodfellow et al., 2014), semantic content (Ramesh et al., 2021)), which are only just beginning to emerge. Algorithms that scale well with the dimensionality of a stimulus can fail to capture high-level stimulus features: for example, PCA-based image models are unable to capture natural image statistics, and do not result in realistic neural receptive field properties (Olshausen and Field, 1996). For these reasons, it is critical that normative plasticity algorithms be able to scale not just to high-dimensional ‘toy’ datasets, but also to complex naturalistic data across sensory modalities. This is a major avenue for improvement: for instance, existing plasticity models have great difficulty scaling to naturalistic image datasets (Bartunov et al., 2018).

Similarly, naturalistic task structures are often much more complex than those used for training general machine learning algorithms, let alone models of normative plasticity (Fig. 2c). In natural environments, rewards are often provided after long sequences of complex actions, supervised feedback is sparse, if present at all, and an organism’s self preservation often requires navigating both uncertainty and complex multi-agent interactions. Modern reinforcement learning algorithms are only just beginning to make progress with some of these difficulties (Kaelbling et al., 1998; Arjona-Medina et al., 2019; Raposo et al., 2021; Hung et al., 2019; Zhang et al., a 2021), but as yet there are no normative plasticity models that describe how any of the human capabilities used to solve these problems could be learned through cellular adaptation (for example, model-based planning (Doll et al., 2012)); similarly, none of these capabilities have been shown to be an emergent consequence of a more basic plasticity process.

### 2.7. Generating testable predictions

Despite the abundance of existing normative theories, very few have been confirmed experimentally, and of those that have received partial confirmation, they are restricted to very specific experimental preparations, for example: fear conditioning in *Aplysia* (Rayport and Schacher, 1986), and reward-based learning in songbird motor systems (Fiete et al., 2007) and in mouse auditory cortex (Froemke et al., 2013; Guo et al., 2019). This relative paucity of validation will not be overcome without a very clear articulation of which features of a normative theory constitute testable predictions, and in what way those predictions disambiguate one theory from its alternatives.

Many existing features of normative theories would be fatal to those theories if proven not to hold in biology. Some examples include: weight symmetry, reward modulation of plasticity, differential roles (and plasticity rules) for apical and basal synapses, and the existence of eligibility traces for temporal credit assignment. However, these individual features, if *proven* to hold, would eliminate alternative theories to highly variable degrees. Most, if not all models could accommodate weight symmetry, several distinct models predict reward modulation of plasticity either through precise credit assignment or global neurotransmitter delivery (Murray, 2019; Williams, 1992; Bellec et al., 2020; Roth et al., 2018), and several distinct supervised and unsupervised models predict different types of signaling and plasticity at apical and basal synapses on pyramidal neurons (Urbanczik and Senn, 2014; Payeur et al., 2021; Bredenberg et al., 2021; Körding and König, 2001; Schiess et al., 2016; Sacramento et al., 2017; Guerguiev et al., 2017; Richards and Lillicrap, 2019), while nearly all models capable of temporal credit assignment assume some form of synaptic eligibility trace (Bellec et al., 2020; Marschall et al., 2020; Murray, 2019; Miconi, 2017; Roth et al., 2018). It is intuitively clear that for any given normative theory of synaptic plasticity, there exist an infinite number of infinitesimal perturbations to that theory that would be impossible to disambiguate experimentally. Further, there are many features of normative theories that would be fatal if proven not to hold, but are completely unclear how to test experimentally.

**Figure 3:**
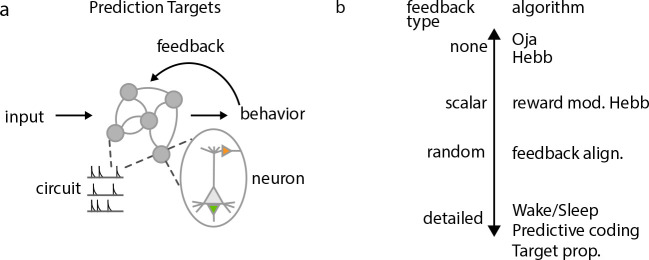
Testing normative theories. **a.** Normative plasticity theories can be assessed through four different experimental lenses centered on individual neurons, circuits of collectively recorded neurons, the training signals delivered to a circuit, and the organism’s overall behavior over the course of learning. **b.** Different normative plasticity theories postulate different levels of detail for the feedback signals received by individual neurons.

The most useful predictions are those that are fatal to the theory if proven false, are clearly testable, and disambiguate the theory from the greatest number of alternative theories. It may be that a collection of predictions is required to completely isolate one individual normative theory from closely related models, which suggests that articulating where particular models lie within a taxonomy of predictions is the most useful way to narrow down the field of possible models. Testable predictions can be defined in terms of several different experimental lenses, of which we isolate four: experiments examining individual neurons or synapses, populations of neurons, the feedback mechanisms that shape learning in neural circuits, or learning at a behavioral level (Fig. 3a). Accurately distinguishing one mechanism from another will likely require a synthesis of experiments spanning all four lenses.

#### Individual neurons.

Experiments that focus on individual neurons, including paired-pulse stimulation (Markram et al., 1997), mechanistic characterizations of plasticity (Graupner and Brunel, 2010), pharmacological explorations of neuromodulators that induce or modify plasticity (Bear and Singer, 1986; Reynolds and Wickens, 2002; Froemke et al., 2007; Gu and Singer, 1995), and characterization of local dendritic or microcircuit properties mediating plasticity (Froemke et al., 2005; Letzkus et al., 2006; Sjöström and Häusser, 2006) form the bulk of the classical literature underlying phenomenological and mechanistic modeling. These studies characterize what information is locally available at synapses and what can be done with that information, as well as which properties of cells can be altered in an experience-dependent fashion.

Existing normative theories differ in the nature of their predictions for plasticity at individual neurons. Reward-modulated Hebbian theories *require* feedback information be delivered by a neuromodulator like dopamine, serotonin, or acetylcholine (Frémaux and Gerstner, 2016) and that this feedback modulates plasticity at the local synapse by changing the magnitude or sign of plasticity depending on the strength of feedback. In contrast, some unsupervised normative theories require no feedback modulation of plasticity (Pehlevan et al., 2015, 2017), and others argue that detailed feedback information arrives at the apical dendritic arbors of pyramidal neurons to modulate plasticity, which is also partially supported in the hippocampus (Bittner et al., 2015, 2017) and cortex (Larkum et al., 1999; Letzkus et al., 2006; Froemke et al., 2005; Sjöström and Häusser, 2006).

Independent of the exact feedback mechanism, models differ in how temporal associations are formed. Algorithms related to REINFORCE assume that local synaptic eligibility traces integrate over time fluctuations in coactivity of the post- and pre-synaptic neuron local to a synapse. These postulated eligibility traces are stochastic, summing Gaussian fluctuations in activity (Miconi, 2017) that consequently produce temporal profiles similar to Brownian motion. In contrast, methods based on approximations to real-time recurrent learning propose eligibility traces that are deterministic records of coactivity whose time constants are directly connected to the dynamics of the neuron itself (Bellec et al., 2020), while other hybrid approaches predict eligibility traces which are deterministic but are related more to predicted task timescale than the dynamics of the cell (Roth et al., 2018). Though there do exist known cellular processes that naturally track coactivity, like NMDA receptors (Bi and Poo, 1998), and that store traces of this coactivity longitudinally, like CaMKII (Graupner and Brunel, 2010), much work remains to be done to analyze how the properties of these known biophysical quantities relate to the predictions of various normative theories, and whether there are other biological alternatives. Other algorithms have different predictions at a microcircuit, rather than at an individual neuron level. Impression learning, for instance, suggests that a population of inhibitory interneurons could gate the influence of apical and basal dendritic inputs to the activity of pyramidal neurons (Bredenberg et al., 2021), and some forms of predictive coding propose that top-down error signals are partially computed by local inhibitory interneurons. Therefore, to completely distinguish different theories, it may be necessary to analyze the connectivity and plasticity between small groups of different cell types.

In sum, experiments at the level of individual neurons or local microcircuits potentially have a great deal of power to identify whether a particular neural circuit is implementing any of a collection of hypothesized normative models of plasticity. It is an advantage that these methods can identify the adaptive capabilities of individual neurons and synapses, but these methods are also limited in their ability to simultaneously observe the adaptation of many neurons in a circuit. Normativity is inherently concerned with the value of plasticity for perception and behavior, and as we will see in subsequent sections, experiments targeting larger populations of neurons will be necessary to distinguish certain features of these theories.

#### Neural circuits.

Determining how circuits encode environmental information and affect motor actions by an animal cannot be assessed by looking at single neurons, and by extension, analyzing how these properties change over time requires methods that record large groups of neurons, such as 2 photon calcium imaging, multielectrode recordings, fMRI, EEG, and MEG, as well as methods that manipulate large populations, like optogenetic (Rajasethupathy et al., 2016) stimulation. The benefits of these recording techniques for testing normative plasticity models, though less practiced compared to individual neuron studies, are manyfold. One of the challenges for characterizing a circuit with a normative plasticity model is selecting an appropriate objective function. Determining which objective fits best can partly be determined by philosophical considerations (Section 2.1), but empirical validation is a far more rigorous test. For instance, one can establish that explicit reward modifies a neural representation to improve coding of task-relevant variables (Froemke et al., 2013). Another line of approaches trains neural networks on a battery of objectives, and determines which objective produces the closest correspondence between model neurons and neurons recorded brain in a variety of areas in the ventral (Yamins et al., 2014; Yamins and DiCarlo, 2016) and dorsal (Mineault et al., 2021) visual streams, as well as recently in auditory cortex (Kell et al., 2018) and medial entorhinal cortex (Nayebi et al., 2021). Oftentimes, changes in artificial neural network activity throughout time are sufficient to determine the objective optimized by the network as well as its learning algorithm (Nayebi et al., 2020), an approach which could also potentially be applied to recorded neural activity over learning.

Beyond narrowing down the objective function, recording from populations can establish features of neural learning that normative models must account for. For instance, in biofeedback training settings, animals can selectively control the firing rates of individual neurons to satisfy arbitrary experimental conditions for reward (Fetz, 2007), suggesting the existence of highly flexible credit assignment systems in the brain, which are not constrained by evolutionary predetermination of the function of neural circuits^2^. Further, circuit recordings could in principle test predictions about how neural circuits should function in situations that do not specifically involve learning. For instance, the Wake-Sleep algorithm (Dayan et al., 1995) (Appendix D) proposes that neural circuits should spend extended periods of time (e.g. during dreaming) generating similar activity patterns to those evoked by natural stimulus sequences, whereas impression learning proposes that similar hallucinatory states could be induced by experimentally increasing the influence of apical dendrites on pyramidal neuron activity (Bredenberg et al., 2021). An alternative learning algorithm based on generative adversarial networks proposes that during sleep networks rehearse corrupted versions of recent waking experiences (Deperrois et al., 2021). There is plenty of room for experiments to more clearly map predictions and components of these models onto well documented neural phenomena, such as sleep or potentially replay phenomena (Girardeau et al., 2009; Eschenko et al., 2008). Because circuit recording and manipulation methods often sacrifice temporal resolution (Hong and Lieber, 2019), and have difficulty inferring biophysical properties of individual synapses and cells, these methods are best used in concert with single neuron studies to jointly tease apart the multi-level predictions of various normative models.

#### Feedback mechanisms.

One of the best ways to distinguish normative plasticity algorithms is on the basis of the nature of their feedback mechanisms (Fig. 3b). Though some unsupervised algorithms, like Oja’s rule propose that no feedback is necessary to perform meaningful learning, no current normative theories propose any form of supervised or reinforcement learning that does not require *some* form of top-down feedback. However, across these models, the level of precision of feedback varies considerably. The simplest feedback is scalar, conveying reward (Williams, 1992), state fluctuation (Payeur et al., 2021), or context (e.g. saccade (Illing et al., 2021) or attention (Roelfsema and Ooyen, 2005; Pozzi et al., 2020)) information. Beyond this, the space of proposed mechanisms expands considerably: backpropagation approximations like feedback alignment (Lillicrap et al., 2016) and random-feedback online learning (RFLO) (Murray, 2019) propose random feedback between layers of neurons can provide a sufficient learning signal, whereas algorithms based on control theory propose that low-rank or partially random projections carrying supervised error signals are sufficient (Gilra and Gerstner, 2017; Alemi et al., 2018). Other algorithms propose even more detailed feedback, with individual neurons receiving precise, carefully adapted projections carrying learning-related information. These algorithms propose that top-down projections to apical dendrites (Urbanczik and Senn, 2014) or local interneurons neurons (Bastos et al., 2012) perform spatial credit assignment, but the nature of this signal can differ considerably across different algorithms. It could be a supervised target, carrying information about what the neuron state ‘should’ be to achieve a goal (Guerguiev et al., 2017; Payeur et al., 2021), or it could be a prediction of the future state of the neuron (Bredenberg et al., 2020).

Each of these different possibilities is theoretically testable, if the focus is shifted to the postulated feedback mechanism, instead of the circuit undergoing learning. However, so far the different mechanisms have received only partial support. For example, acetylcholine projections to auditory cortex that modulate perceptual learning (Froemke et al., 2013) display a diversity of responses related to both reward and attention (Hangya et al., 2015), which adapt over the course of learning in concert with auditory cortex (Guo et al., 2019). This suggests that while traditional models of reward-modulated Hebbian plasticity may be correct to a first approximation, a more detailed study of the adaptive capabilities of neuromodulatory centers may be necessary to update the theories.

While a growing number of studies indicate that projections to apical synapses of pyramidal neurons *do* play a role in inducing plasticity, and that these projections themselves are also plastic (i.e. nonrandom) (Bittner et al., 2015, 2017), very little is known about the *nature* of the signal—a critical component for distinguishing several different theories. In the visual system, presentation of unfamiliar images without any form or reward or supervision can modify both apical and basal dendrites throughout time (Gillon et al., 2021), and in the hippocampus, apical input to CA1 pyramidal neurons while animals acclimatize to new spatial environments is sufficient to induce synaptic plasticity (Bittner et al., 2015, 2017). These two examples support a form of *unsupervised* learning, but evidence for supervised or reinforcement learning signals propagated through apical dendritic synapses is currently lacking. Beyond the cerebellar system, where climbing fiber pathways may carry explicit motor error signals used for plasticity (Gao et al., 2012; Bouvier et al., 2018), evidence for detailed supervised feedback is limited. In sum, beyond single neurons, or even populations recorded by traditional techniques, targeted focus on the learning feedback signals received by a population shows promise to rule out algorithms on the basis of their feedback and objective function.

#### Behavior.

In much the same way that psychophysical studies of human or animal responses define constraints on what the brain’s perceptual systems are capable of, behavioral studies of learning can do quite a lot to describe the range of phenomena that a model of learning must be able to capture, from operant conditioning (Niv, 2009), to model-based learning (Doll et al., 2012), rapid language learning (Heibeck and Markman, 1987), unsupervised sensory development (Wiesel and Hubel, 1963), or consolidation effects (Stickgold, 2005). Behavioral studies can also outline key limitations in learning, which are perhaps reflective of the brain’s learning algorithms, including the brain’s failure to perform certain types of adaptation after critical periods of plasticity (Wiesel and Hubel, 1963), and the brain’s unexpected inability to learn multi-context motor movements without explicit motor differences across contexts (Sheahan et al., 2016).

These existing experimental results stand as (often unmet) targets for normative theories of plasticity, but in addition, normative theories themselves suggest further studies that may test their predictions. In particular, manipulation of learning mechanisms may have predictable effects on animals’ behavior, as seen when acetylcholine receptor blockage in mouse auditory cortex prevented reward-based learning in animals (Guo et al., 2019), and nucleus basalis stimulation during tone perception longitudinally improved animals’ discrimination of that tone (Froemke et al., 2013). Other algorithms have as-yet untested predictions for behavior: for instance, experimentally increasing the influence of top-down projections should bias behavior towards commonly-occurring sensory stimuli according to both predictive coding (Rao and Ballard, 1999; Friston, 2010) and impression learning (Bredenberg et al., 2021). For other detailed feedback algorithms (Fig. 3b), manipulating top-down projections may disrupt learning, but would have a much more unstructured deleterious effect on perceptual behavior.

As shown, each experimental lens has its own advantages and disadvantages. Single-neuron studies are excellent for identifying the locally-available variables that affect plasticity, circuit-level studies can help narrow down the objectives that shape neural responses and identify traces of offline learning, studies of feedback mechanisms can distinguish between different algorithms that postulate different degrees of precision in their feedback and in complexity of the teaching signal, and studies of behavior can place boundaries on what can be learned, as well as serve as a readout for manipulations of the mechanisms underlying learning. Each focus alone is insufficient to distinguish between all existing normative models, but in concert they show promise for identifying the neural substrates of adaptation.

## 3. Conclusions

Normative plasticity models are compelling because of their potential to connect our brains’ capacity for adaptation to their constituent synaptic modifications. Generating good theories is a critical part of the scientific process, but finding ways to close the loop by testing key predictions of new normative models has proved extraordinarily difficult: in this perspective we have illustrated the sources of this difficulty.

The core of a normative plasticity model is its plasticity rule, which dictates how a model synapse modifies its strength. To be a normative model—to explain why the plasticity mechanism is important for the organism—there must be a concrete demonstration that this plasticity rule supports adaptation critical for system-wide goals like processing sensory signals or obtaining rewards (Section 2.1). However, this system-wide goal must be achieved using only *local* information (Section 2.2). These two needs of a normative plasticity model are the fundamental source of tension: it is very difficult to demonstrate that a proposed plasticity rule is both local *and* optimizes a system-wide objective (Appendix B). Insufficient or partial resolution of this fundamental tension produces normative models that struggle to map accurately onto neural hardware (Section 2.3) or handle complex temporal stimuli and tasks online (Sections 2.4–2.6). To provide a case study of how our desiderata come to be satisfied (or not) in practice, we have included tutorials for both REINFORCE and the Wake-Sleep algorithm in Appendices C and D. These tutorials are by no means a complete introduction to the field, but will hopefully serve as a solid foothold for analyzing modern normative plasticity models.

Even satisfying the aforementioned desiderata, much work remains to delineate which tests would most clearly distinguish a normative model from its alternatives in a biological system. In this review, we have organized emerging theories according to how they satisfy and improve upon our desiderata (Table 1), as well as by how they can be tested (Section 2.7), with the view that this organization will provide avenues for both experimental and theoretical neuroscientists to bring normative plasticity models closer to biology. Even if existing algorithms prove not to be implemented exactly in the brain, they undoubtedly provide key insights into how local synaptic modifications can produce valuable improvements in both behavior and perception for an organism. It seems sensible to use these algorithms as a springboard to produce more biologically realistic and powerful theories.

Beyond improving normative theories with respect to our desiderata, there are several incredible opportunities for actually testing their implementation in biology (Section 2.7). Most current theoretical studies of reward-modulated Hebbian plasticity focus on dopamine-modulated motor learning in monkeys and songbirds (Fiete et al., 2007; Legenstein et al., 2010), but there are *many* neuromodulatory systems that have been linked to learning in experiments, including serotonin-modulated fear conditioning in the amygdala (Lesch and Waider, 2012), as well as acetylcholine-modulated reward learning and oxytocin-modulated social learning in mouse auditory cortex (Guo et al., 2019; Froemke et al., 2013). Further, several experimental preparations examine the relationship between pyramidal neurons’ apical and basal dendritic activity and plasticity, in both the hippocampus (Bittner et al., 2015, 2017) and visual cortex (Gillon et al., 2021; Froemke et al., 2005; Letzkus et al., 2006; Sjöström and Häusser, 2006). These could test at the level of individual neurons, circuits, behavior, and the feedback mechanisms that support plasticity, which of the many alternative normative theories underlie animals’ learning.

As the diversity of aforementioned experimental preparations suggests, there are increasingly strong arguments for several fundamentally different plasticity algorithms instantiated in different areas of the brain and across different organisms, subserving different functions. It is quite likely that many plasticity mechanisms work in concert to produce learning as it manifests in our perception and behavior. It is our belief that well-articulated normative theories can serve as the building blocks of a conceptual framework that tames this diversity and allows us to understand the brain’s tremendous capacity for adaptation.

## Supplementary Material

Supplement 1

## Figures and Tables

**Table 1:** Summarizing progress on the desiderata. A **✓** indicates that an algorithm has been demonstrated to satisfy a particular desideratum in at least one study, whereas an **✗** indicates that it has not been demonstrated. If the demonstrating study is an improvement on the seminal work or is a new model, we provide a citation; reference numbering used for brevity: Asterisks (*) indicate that results have only been shown by simulation, and lack mathematical support. U, S, and R indicate whether a given algorithm supports unsupervised, supervised, or reinforcement learning, respectively

Algorithm	Dec. Loss	Local	Arch.	Time	Online	Scalable
Backpropagation (Wer74)	U/S/R (Wil92)	**✗**	**✓** (Lee16)	**✓** (Wer90)	**✗**	**✓**
REINFORCE (Wil92)	U/S/R	**✓**	**✓**	**✓** (Mic17)	**✓**	**✗** (Wer03)
Oja (Oja82)	U	**✓**	**✗**	**✗**	**✓**	**✓**
Predictive Coding (Rao99)	U/S (Whi17)	**✓**	**✗**	**✓** (Fri09)	**✓**	**✓**
Wake-Sleep (Day95)	U	**✓**	**✓** (Day96)	**✓** (Day96)	**✓** (Bre21)	**✓**
Approx. Backprop. (Lil16) (Akr19)	U/S*	**✓**	**✓** (Bel20)	**✓** (Mur19) (Bel20)	**✓** (Mur19) (Bel20)	**✓**
Equilibrium Prop. (Sce17)	U/S	**✓**	**✗**	**✗**	**✓** (Ern20)	**✓** (Lab21)
Target Prop. (Ben14)	U/S	**✓**	**✓**	**✓** (Man20)	**✗**	**✓** (Lee15)

## Footnotes

1 Some objectives (like reward functions) are best thought of as being maximized rather than minimized. Without loss of generality, in such cases we can minimize the negative reward function.

2 This is a challenge for normative plasticity models that predefine the outputs of the circuit and approximately backpropagate errors from these outputs.
