# Supplementary material for: Desiderata for normative models of synaptic plasticity
Source: ArXiv. 2023 Aug 9:arXiv:2308.04988v1. Preprint. [Version 1] (PMC10441445)
Supplement: Supplement 1 [file NIHPP2308.04988v1-supplement-1.pdf]

# A The unidentifiability of an objective

In this section we illustrate why the choice of objective function for a normative plasticity model is never uniquely determined by data. We will consider two situations: the system has already settled to its optimal setting of its weights, $\mathbf{W}^*$, and in the second we are able to observe the system's plasticity update $\Delta\mathbf{W}$.

## A.1 Unidentifiability based on an optimum

Suppose that some setting of synaptic weights $\mathbf{W}^*$ minimizes an objective function $\mathcal{L}$, i.e. $\mathcal{L}(\mathbf{W}^*) \leq \mathcal{L}(\mathbf{W})\ \forall \mathbf{W}$. We might be tempted to argue that because $\mathbf{W}^*$ minimizes $\mathcal{L}$, $\mathcal{L}$ must be *the* objective that the system is minimizing. However, there are an infinite variety of alternative objectives that share the same minimum. To see this, take a new objective $\tilde{\mathcal{L}} = \sigma\left(\mathcal{L}(\mathbf{W})\right)$ for any differentiable, monotonically increasing function $\sigma(\cdot)$. Then we have:

$$\begin{aligned} &\mathcal{L}(\mathbf{W}^*) \leq \mathcal{L}(\mathbf{W})\ \forall \mathbf{W} && (4)\\ \Rightarrow &\sigma\left(\mathcal{L}(\mathbf{W}^*)\right) \leq \sigma\left(\mathcal{L}(\mathbf{W})\right)\ \forall \mathbf{W} && (5)\\ \Rightarrow &\tilde{\mathcal{L}}(\mathbf{W}^*) \leq \tilde{\mathcal{L}}(\mathbf{W})\ \forall \mathbf{W}, && (6) \end{aligned}$$

where the second equality follows from the order preservation property of $\sigma(\cdot)$. This means that $\mathbf{W}^*$ also minimizes $\tilde{\mathcal{L}}$, i.e. we will be unable to arbitrate between whether the system is 'attempting' to minimize $\tilde{\mathcal{L}}$ or $\mathcal{L}$ on the basis of the optimized network state given by $\mathbf{W}^*$.

## A.2 Unidentifiability based on an update rule

Suppose instead that we were able to observe the adaptive plasticity mechanism of a system, and were able to verify that it really does decrease an objective function $\mathcal{L}$, i.e. by Eq. 3,

$$\frac{d\mathcal{L}}{d\mathbf{W}}(\mathbf{W})^T \Delta\mathbf{W} \leq 0\ \forall \mathbf{W}. \tag{7}$$

We might now be tempted to argue that, by observing the plasticity rule itself, $\Delta\mathbf{W}$, we will be more able to assert that the system, by virtue of consistently decreasing $\mathcal{L}$, is 'attempting' to minimize $\mathcal{L}$. However, the *exact same* family of alternative objectives will also be minimized ($\tilde{\mathcal{L}} = \sigma\left(\mathcal{L}(\mathbf{W})\right)$ for any differentiable, monotonically increasing function $\sigma(\cdot)$). To see this, we observe:

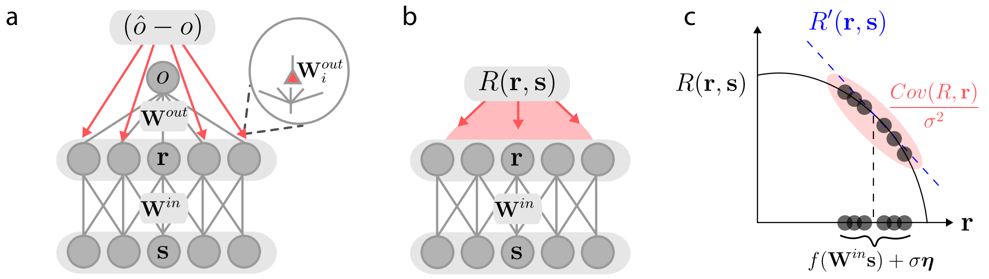


Figure S1: **Weight transport and REINFORCE. a.** Traditional gradient descent propagates a credit assignment signal $(\hat{o}-o)\mathbf{W}_i^{out}$ to each neuron $\mathbf{r}_i$. How this pathway could have access to $\mathbf{W}_i^{out}$ is unclear: this is the 'weight transport' problem. **b.** REINFORCE resolves the weight transport problem by projecting a scalar reward signal $R(\mathbf{r},\mathbf{s})$ to all synapses. **c.** By correlating this reward with fluctuations in neural activity, neurons can approximate the true gradient.

$$\frac{d\mathcal{L}}{d\mathbf{W}}(\mathbf{W})^T\Delta\mathbf{W} \leq 0 \ \forall\mathbf{W} \tag{8}$$

$$\Rightarrow \frac{d\sigma(\mathcal{L}(\mathbf{W}))}{d\mathcal{L}(\mathbf{W})}\frac{d\mathcal{L}}{d\mathbf{W}}(\mathbf{W})^T\Delta\mathbf{W} \leq 0 \ \forall\mathbf{W} \tag{9}$$

$$\Rightarrow \frac{d\tilde{\mathcal{L}}}{d\mathbf{W}}(\mathbf{W})^T\Delta\mathbf{W} \leq 0 \ \forall\mathbf{W}, \tag{10}$$

where the first implication follows from the fact that $\sigma(\cdot)$ is differentiable and increasing (it has strictly positive derivative), and the second implication follows from the chain rule. This implies that plasticity rules ($\Delta\mathbf{W}$) and trained neural circuits ($\mathbf{W}^*$) can at most partially constrain the space of viable objective functions the system could be minimizing.

# B Why can't the brain do explicit gradient descent?

We have provided one surefire way to decrease an objective function by modifying the parameters of a neural network—'simply' take small steps in the direction of the gradient of the loss (Section 2.1). To appreciate the challenges faced by theories of normative plasticity, it's important to understand why a biological system *could not* do this: in this section we will provide a simplified argument as to why gradient descent within multilayer neural networks produces *nonlocal* parameter updates, thus failing our most critical desideratum for a normative plasticity theory (Section 2.2). More detailed arguments for multilayer neural networks can be found here (Lillicrap et al., 2020), and descriptions of why gradient descent becomes even more implausible for recurrent neural networks trained with either backpropagation through time

(Werbos, 1990) or real-time recurrent learning (Williams and Zipser, 1989) can be found here (Marschall et al., 2020).

The 'weight transport problem' is the most basic reason that gradient descent is implausible for neural networks. Suppose that we have a stimulus-dependent network response, $\mathbf{r}(\mathbf{W}^{in}) = f(\mathbf{W}^{in}\mathbf{s})$, where $\mathbf{r}$ is an $N \times 1$ vector, and $\mathbf{W}^{in}$ is an $N \times N^s$ weight matrix mapping stimuli $\mathbf{s}$ into responses after a pointwise nonlinearity $f(\cdot)$. This network response is decoded into a network output, $o(\mathbf{W}^{in}, \mathbf{s}) = \mathbf{W}^{out}\mathbf{r}(\mathbf{W}^{in})$, where $\mathbf{W}^{out}$ is a $1 \times N$ vector mapping network responses into a scalar output. Now suppose for simplicity that our loss for a single stimulus example is given by:

$$\mathcal{L} = \frac{1}{2}\left(\hat{o} - o(\mathbf{W}^{in}, \mathbf{s})\right)^2. \tag{11}$$

This objective is trying to bring the stimulus-dependent network response $o(\mathbf{W}^{in}, \mathbf{s})$ close to the target output $\hat{o}$, and is zero if and only if $o = \hat{o}$. A reasonable hypothesis would be that the gradient of this objective function with respect to a synaptic weight, $\mathbf{W}^{in}_{ij}$, will produce a parameter update that is local: we will see that this is not true. Taking the gradient, we have:

$$\frac{d}{d\mathbf{W}^{in}_{ij}}\mathcal{L} = \frac{1}{2}\frac{d}{d\mathbf{W}^{in}_{ij}}\left(\hat{o} - o(\mathbf{W}^{in}, \mathbf{s})\right)^2 \tag{12}$$

$$= (\hat{o} - o)\,\frac{d}{d\mathbf{W}^{in}_{ij}}o(\mathbf{W}^{in}, \mathbf{s}) \tag{13}$$

$$= (\hat{o} - o)\,\mathbf{W}^{out}_i\frac{d}{d\mathbf{W}^{in}_{ij}}f_i(\mathbf{W}^{in}\mathbf{s}) \tag{14}$$

$$= (\hat{o} - o)\,\mathbf{W}^{out}_i f_i'(\mathbf{W}^{in}\mathbf{s})\mathbf{s}_j. \tag{15}$$

Breaking down this final update, we can see three terms: an error, $(\hat{o} - o)$, the neuron's *output weight* $\mathbf{W}^{out}_i$, and an approximately Hebbian term $f_i'(\mathbf{W}^{in}\mathbf{s})\mathbf{s}_j$, which requires only a combination of pre- and post-synaptic activity. One might be tempted to organize the plasticity rule into a error feedback signal received by the neuron, scaled by a neuron-specific synaptic weight $\mathbf{W}^{out}_i$, and then combined with Hebbian coactivity to produce a synaptic update (Fig. S1a). This would have the form of a three-factor plasticity rule (Frémaux and Gerstner, 2016), combining weighted feedback with pre- and post-synaptic activity. However, the weight transport problem is as follows: $\mathbf{W}^{out}_i$ provides the strength of a synapse in the *feedforward* pathway—how could it possibly come to be that a feedback learning pathway would have access to the *same* synaptic weight? The answer is that there is no evidence for such a system of weight sharing across feedforward and feedback pathways in the brain, though there are many hypotheses about how such a system could, in theory, be approximated by a normative plasticity algorithm. This problem becomes more pronounced in multilayer networks, where the error signal must be propagated through many interconnected connectivity layers.

It is also worth noting two key differentiability assumptions inherent to this approach. For one, we assume not only that the loss function $\mathcal{L}$ is differentiable, but that some 'error calculating' part of the brain does differentiate it. This requires knowledge of what the desired network output should be $\hat{o}$, which for many real-world tasks is not possible. Second, we assume that the network activation function $f(\cdot)$ is differentiable. Since neurons typically emit binary spikes, this differentiability assumption is not necessarily valid, though several modern methods have circumvented this problem by using either stochastic neuron models (Williams, 1992; Dayan and Hinton, 1996) or by using clever optimization tricks (Bellec et al., 2020). In subsequent sections, we will outline two canonical algorithms that employ clever tricks to circumvent the weight transport problem.

# C REINFORCE

In this section, we will provide a mathematical tutorial on the REINFORCE learning algorithm (Williams, 1992), which is a mechanism for updating the parameters in a stochastic neural network for reinforcement learning objective functions. Its chief advantages are twofold: first, it only requires you to be able to evaluate an objective function (i.e. the reward received on any given trial), not the gradient of the objective function with respect to the parameters (Fig. S1b). This is very useful in situations in which the relationship between rewards and network outputs is not clear to an agent, as would be the case in many reinforcement learning scenarios. Second, under a broad range of biologically reasonable assumptions about a neural network architecture, the parameter updates produced by this algorithm are 'local,' meaning the information required for a parameter update would reasonably be available to a synapse in the brain. This algorithm produces updates that are within the class of 'reward-modulated Hebbian plasticity rules.' The chief disadvantage of this algorithm is its comparative data-inefficiency relative to backpropagation. In practice, far more data samples (or equivalently, much lower learning rates) will be required to produce the same improvements in performance compared to backpropagation (Werfel et al., 2003).

The REINFORCE algorithm and minor variations appear in different fields with different names. It is useful to keep track of these alternative names, because they all use roughly the same derivation, with some improvements or field-specific modifications. In machine learning, the algorithm is often referred to as *node perturbation* (Richards et al., 2019; Lillicrap et al., 2020; Werfel et al., 2003), because it involves correlating fluctuations in neuron (node) activity with reward signals. In computational neuroscience, it is sometimes called *3-factor* or *reward-modulated Hebbian* plasticity (Frémaux and Gerstner, 2016), though REINFORCE is only one of several algorithms referred to by these blanket terms. In reinforcement learning, REINFORCE is often treated as a member of the more general class of *policy gradient* (Sutton and Barto, 2018) methods, which can be used to train any parameterized stochastic agent through reinforcement. Policy gradient methods need not commit to a neural network architecture, and are consequently not always local. Lastly, very

similar methods are used for fitting variational Bayesian models, and are in these contexts referred to as either *black box variational inference* (Ranganath et al., 2014) or *neural variational inference* (Mnih and Gregor, 2014).

In what follows, we will provide a brief derivation of the REINFORCE learning algorithm for a 1-layer feedforward neural network. We will then discuss the many extensions of the algorithm as well as its strengths and limitations as a normative plasticity model.

## C.1 Network model

Most neural networks used in machine learning are deterministic. However, neurons in biological systems fluctuate across trials and stimulus presentations, so modeling them as stochastic is often more appropriate. It will turn out that these fluctuations can be used to produce parameter updates in a way that a deterministic system could not.

First, we will assume that there are stimuli drawn from some stimulus distribution, $p(\mathbf{s})$, and we will define the neural network response to a given stimulus drawn from this distribution as:

$$\mathbf{r} = f(\mathbf{W}^{in}\mathbf{s}) + \sigma\boldsymbol{\eta}, \tag{16}$$

where the $\boldsymbol{\eta}$ is the source of random fluctuations which, for simplicity, is drawn from a standard normal distribution ($\mathcal{N}(0,1)$). In this equation, $\mathbf{s}$ is an $N_s \times 1$ vector, $\mathbf{W}^{in}$ is an $N_r \times N_s$ matrix, $f(\cdot)$ is the tanh nonlinearity, and $\boldsymbol{\eta}$ is an $N_r \times 1$ vector.

This equation defines a conditional probability distribution, $p(\mathbf{r}|\mathbf{s};\mathbf{W}^{in}) \sim \mathcal{N}(f(\mathbf{W}^{in}\mathbf{s}), \sigma^2)$. There is an interesting point here: neuron activities are now samples from this conditional probability distribution, and so we can study how neurons behave on average by taking expectations over the probability distribution.

For simplicity and clarity we will restrict ourselves to this neural architecture for our derivation, but the basic principles apply more generally to a variety of noise sources and neural architectures (see Section C.5).

## C.2 Defining the objective

We will assume that our goal is to maximize some instantaneous reward $R(\mathbf{r},\mathbf{s})$ on average across many different samples of $R(\mathbf{r},\mathbf{s})$ and $\mathbf{s}$. This allows us to write our objective function $\mathcal{O}(\mathbf{W}^{in})$ as:

$$\mathcal{O}(\mathbf{W}^{in}) = \int R(\mathbf{r},\mathbf{s})p(\mathbf{r}|\mathbf{s};\mathbf{W}^{in})p(\mathbf{s})d\mathbf{r}d\mathbf{s}. \tag{17}$$

In practice, this integral might be analytically impossible to integrate, but we can always approximate it (because it is an expectation) using samples from $p(\mathbf{r}|\mathbf{s};\mathbf{W}^{in})$ and $p(\mathbf{s})$ as an empirical average over $K$ samples $\mathbf{r}_k$ and $\mathbf{s}_k$:

$$\mathcal{O}(\mathbf{W}^{in}) \approx \frac{1}{K}\sum_{k=0}^{K} R(\mathbf{r}^{(k)},\mathbf{s}^{(k)}). \tag{18}$$

Procedurally, this would amount to sampling $\mathbf{s}$ and $\mathbf{r}$ each $K$ times, calculating the reward for each trial, and taking an average.

## C.3 Taking the gradient

Now that we have our objective function, we can evaluate its derivative with respect to a particular synapse $\mathbf{W}^{in}_{ij}$ in the network:

$$\frac{d\mathcal{O}(\mathbf{W}^{in})}{d\mathbf{W}^{in}_{ij}} = \frac{d}{d\mathbf{W}}\int R(\mathbf{r},\mathbf{s})p(\mathbf{r}|\mathbf{s};\mathbf{W}^{in})p(\mathbf{s})d\mathbf{r}d\mathbf{s} \tag{19}$$

$$= \int R(\mathbf{r},\mathbf{s})\left[\frac{d}{d\mathbf{W}^{in}_{ij}}p(\mathbf{r}|\mathbf{s};\mathbf{W}^{in})\right]p(\mathbf{s})d\mathbf{r}d\mathbf{s}. \tag{20}$$

We could theoretically stop here and evaluate $\frac{d}{d\mathbf{W}^{in}_{ij}}p(\mathbf{r}|\mathbf{s};\mathbf{W}^{in})$ explicitly. However, in the same way that we can approximate $\mathcal{O}(\mathbf{W}^{in})$ as an empirical average over samples, we would like to be able to approximate our derivative as an average. To do this requires us to keep our loss in the form of an expectation over $p(\mathbf{r}|\mathbf{s};\mathbf{W}^{in})p(\mathbf{s})$. We notice a convenient identity: $\frac{d}{d\mathbf{W}^{in}_{ij}}p(\mathbf{r}|\mathbf{s};\mathbf{W}^{in}) = \frac{d}{d\mathbf{W}^{in}_{ij}}\exp(\log p(\mathbf{r}|\mathbf{s};\mathbf{W}^{in})) = \left[\frac{d}{d\mathbf{W}^{in}_{ij}}\log p(\mathbf{r}|\mathbf{s};\mathbf{W}^{in})\right]p(\mathbf{r}|\mathbf{s};\mathbf{W}^{in})$, which is a simple application of the chain rule. Inserting this identity into the above equation, we get:

$$\frac{d\mathcal{O}(\mathbf{W}^{in})}{d\mathbf{W}^{in}_{ij}} = \int R(\mathbf{r},\mathbf{s})\left[\frac{d}{d\mathbf{W}^{in}_{ij}}\log p(\mathbf{r}|\mathbf{s};\mathbf{W}^{in})\right]p(\mathbf{r}|\mathbf{s};\mathbf{W}^{in})p(\mathbf{s})d\mathbf{r}d\mathbf{s} \tag{21}$$

$$\approx \frac{1}{K}\sum_{k=0}^{K} R(\mathbf{r}^{(k)},\mathbf{s}^{(k)})\left[\frac{d}{d\mathbf{W}^{in}_{ij}}\log p(\mathbf{r}^{(k)}|\mathbf{s}^{(k)};\mathbf{W}^{in})\right]. \tag{22}$$

Though this is an approximation, we note that by the Law of Large Numbers, we can improve its accuracy arbitrarily by increasing our number of samples $K$. In practice, however, taking $K = 1$ will prove to be the most straightforward way to

get an update that is local in time—although such an update will still on average match the true gradient exactly, its high variance can lead to very inefficient learning.

We have left the derivation completely general up until this point. Different choices of $p(\mathbf{r}|\mathbf{s};\mathbf{W})$ will produce different updates. Our particular choice gives:

$$\frac{d}{d\mathbf{W}_{ij}^{in}} \log p(\mathbf{r}|\mathbf{s};\mathbf{W}^{in}) = \frac{d}{d\mathbf{W}_{ij}^{in}} \sum_{i=0}^{N_r} \frac{1}{2\sigma^2}(\mathbf{r}_i - f_i(\mathbf{W}^{in}\mathbf{s}))^2 + C \tag{23}$$

$$= \frac{1}{\sigma^2} \sum_{n=0}^{N_r} (\mathbf{r}_i - f_i(\mathbf{W}^{in}\mathbf{s})) \frac{df_i(\mathbf{W}\mathbf{s})}{d\mathbf{W}_{ij}^{in}}. \tag{24}$$

For a particular weight $\mathbf{W}_{ij}^{in}$, $\frac{df_l(\mathbf{W}^{in}\mathbf{s})}{d\mathbf{W}_{ij}} = 0$ if $i \neq l$, so we have:

$$\frac{d}{d\mathbf{W}^{in}ij} \log p(\mathbf{r}|\mathbf{s};\mathbf{W}) = \frac{1}{\sigma^2}(\mathbf{r}_i - f_i(\mathbf{W}\mathbf{s}))f_i'(\mathbf{W}\mathbf{s})\mathbf{s}_j. \tag{25}$$

Plugging this equation into Eq. 19 gives the following parameter update:

$$\Delta\mathbf{W}_{ij}^{in} \propto \frac{1}{K}\sum_{k=0}^{K} R(\mathbf{r}^{(k)}, \mathbf{s}^{(k)}) \left[\frac{1}{\sigma^2}(\mathbf{r}_i^{(k)} - f_i(\mathbf{W}^{in}\mathbf{s}^{(k)}))f_i'(\mathbf{W}^{in}\mathbf{s}^{(k)})\mathbf{s}_j^{(k)}\right] \approx \frac{d\mathcal{O}(\mathbf{W}^{in})}{d\mathbf{W}_{ij}^{in}}. \tag{26}$$

If we want to update all of our parameters simultaneously using parallelized matrix operations, we can write this as an outer product:

$$\Delta\mathbf{W}^{in} \propto \frac{1}{K}\sum_{k=0}^{K} R(\mathbf{r}^{(k)}, \mathbf{s}^{(k)}) \left[\frac{1}{\sigma^2}(\mathbf{r}^{(k)} - f(\mathbf{W}^{in}\mathbf{s}^{(k)})) \odot f'(\mathbf{W}^{in}\mathbf{s}^{(k)})\right] \mathbf{s}^{(k)T}, \tag{27}$$

where $\odot$ denotes a Hadamard (elementwise) vector product. Interestingly, the $\frac{1}{\sigma^2}(\mathbf{r} - f(\mathbf{W}^{in}\mathbf{s}))$ term here is exactly equal to $\boldsymbol{\eta}$.

## C.4 Why don't we need the derivative of the loss?

One way of interpreting this parameter update is that neural units are correlating fluctuations in their neural activity with the rewards received to approximate $\frac{dR(\mathbf{r},\mathbf{s})}{d\mathbf{r}}$ (Fig. S1c). To see this, first notice that:

$$\mathbb{E}\left[b\left[\frac{1}{\sigma^2}(\mathbf{r}-f(\mathbf{W}^{in}\mathbf{s}))\odot f'(\mathbf{W}^{in}\mathbf{s})\right]\mathbf{s}^T\right]_{p(\mathbf{r}|\mathbf{s})}=0, \tag{28}$$

for any constant $b$, because $\mathbb{E}\left[\mathbf{r}-f(\mathbf{W}^{in}\mathbf{s})\right]_{p(\mathbf{r}|\mathbf{s})}=0$. If we take $b=\mathbb{E}\left[R(\mathbf{r},\mathbf{s})\right]_{p(\mathbf{r}|\mathbf{s})}$, then we can rewrite the gradient without changing its expected value:

$$\frac{d\mathcal{O}(\mathbf{W}^{in})}{d\mathbf{W}^{in}_{ij}}=\int(R(\mathbf{r},\mathbf{s})-\mathbb{E}\left[R(\mathbf{r},\mathbf{s})\right]_{p(\mathbf{r}|\mathbf{s})})\left[\frac{1}{\sigma^2}(\mathbf{r}_i-f_i(\mathbf{W}^{in}\mathbf{s}))f_i'(\mathbf{W}^{in}\mathbf{s})\mathbf{s}_j\right]p(\mathbf{r}|\mathbf{s};\mathbf{W}^{in})p(\mathbf{s})d\mathbf{r}d\mathbf{s} \tag{29}$$

$$=\int\frac{1}{\sigma^2}Cov(R(\mathbf{r},\mathbf{s}),\mathbf{r}_i)\left[f_i'(\mathbf{W}^{in}\mathbf{s})\mathbf{s}_j\right]p(\mathbf{s})d\mathbf{s}, \tag{30}$$

where $Cov(R(\mathbf{r},\mathbf{s}),\mathbf{r}_i)=\int(R-\mathbb{E}\left[R\right]_{p(\mathbf{r}|\mathbf{s})})(\mathbf{r}_i-\mathbb{E}\left[\mathbf{r}_i\right]_{p(\mathbf{r}|\mathbf{s})})p(\mathbf{r}|\mathbf{s})d\mathbf{r}$ is the stimulus-conditioned covariance between network firing rates and reward. The sample-based parameter update is therefore using the fluctuations in neural activity to compute this covariance.

## C.5 Assessing REINFORCE

Now that we have derived REINFORCE, we can examine its qualities as a normative plasticity theory. First, we ask: is this algorithm 'local' (Section 2.2)? The gradient for a particular synapse, $\frac{d\mathcal{O}(\mathbf{W}^{in})}{d\mathbf{W}^{in}_{ij}}$ can be approximated with samples in an environment with stimuli $\mathbf{s}$, firing rates $\mathbf{r}$, and rewards $R(\mathbf{r},\mathbf{s})$ by $R(\mathbf{r},\mathbf{s})\left[\frac{1}{\sigma^2}(\mathbf{r}_i-f_i(\mathbf{W}^{in}\mathbf{s}))f_i'(\mathbf{W}^{in}\mathbf{s})\mathbf{s}_j\right]$. To decide whether this could be a plasticity rule implemented (or more realistically, approximated) by a biological system, we need to think about what pieces of information a synapse would have to have available.

First, the synapse needs $\mathbf{s}_j$, which amounts to just the presynaptic input, a common feature of any Hebbian synaptic plasticity rule. Second, the synapse needs $\frac{1}{\sigma^2}(\mathbf{r}_i-f_i(\mathbf{W}^{in}\mathbf{s}))f_i'(\mathbf{W}^{in}\mathbf{s})$. $\frac{1}{\sigma^2}$ is a constant, and so can be absorbed into the learning rate. $\mathbf{r}_i$ is the postsynaptic firing rate, which is also a common feature of any Hebbian plasticity rule. $(\mathbf{W}^{in}\mathbf{s})_i$ is the current injected into the postsynaptic neuron, and $f_i(\cdot)$ and $f_i'(\cdot)$ are both monotonic functions of this current, so it is quite conceivable that these values could be approximated by a biochemical process. Third, every synapse needs access to the scalar reward value received on a given trial, $R(\mathbf{r},\mathbf{s})$. This is the most 'nonlocal' information involved in the parameter update, however, there exist many theories about how neuromodulatory systems in the brain can deliver information about reward diffusely to many synapses and induce plasticity (Section 2.2).

Now, we have already demonstrated that REINFORCE is able to perform approximate gradient descent for reinforcement learning objective functions—this in itself makes the algorithm very promising as a normative plasticity model (Section 2.1).

Its chief advantage is that it does not require detailed knowledge of the reward function $R(\mathbf{r}, \mathbf{s})$ (i.e. how to differentiate it), which means that an animal could simply receive a reward from its environment, and relay that reward signal diffusely to its synapses. However, this also restricts the types of objectives that could plausibly be learned by a neural system. Unsupervised learning objectives like the ELBO require detailed knowledge of every neural activity of every neuron in the circuit in order to be calculable (Appendix D), and there is no evidence for downstream neural circuits that perform such calculations. Therefore, even though in principle REINFORCE can be used to train a neural network on *any* objective, explicit reinforcement is much more plausible than other alternatives.

We have only provided a derivation for a single-layer rate-based neural network with additive Gaussian noise, but REINFORCE extends quite readily to multi-layer (Williams, 1992), spiking (Frémaux et al., 2013), and recurrent networks (Miconi, 2017) without any loss of locality. This indicates that the algorithm is both architecture-general (Section 2.3) and can handle temporal environmental structure (Section 2.4). Further, because a weight update can be calculated in a single trial, animals could use it to learn online (Section 2.5). The biggest point of failure for REINFORCE is that it scales poorly with high complexity in stimuli or task, large numbers of neurons, or prolonged delays in receipt of reward (Werfel et al., 2003; Fiete, 2004; Bredenberg et al., 2021). The greater the number of neurons that contribute to reward and the higher the complexity of the reward function, the harder it becomes to estimate the correlation between a single neuron and reward, which is a prerequisite for the algorithm's function. Thus, though the algorithm is an unbiased estimator of the gradient, it can still be so variable an estimate as to be effectively useless in complex contexts. This suggests that if animals exploit the principles of REINFORCE to update synapses, it is likely an approach paired with other algorithms, or hybridized in a way that allows for better scalability.

The last way to assess REINFORCE is on the basis of how it can be tested (Section 2.7). The simplest way to test this algorithm is by examining whether scalar reward-like signals (i.e. $R(\mathbf{r}, \mathbf{s})$) have a multiplicative effect on local plasticity in a circuit. At a single-neuron level this corresponds to identifying neuromodulators that affect plasticity. At a feedback level this corresponds to identifying neuromodulatory systems that project to the circuit in question, and observing whether their stimulation or silencing improves or blocks circuit-level plasticity or behavioral learning performance, respectively. These steps do not identify REINFORCE as the only possibility, but it narrows down the field of possibilities considerably, removing all candidate algorithms that either do not require any feedback, or that require more detailed feedback signals (Fig. 3a).

# D Wake-Sleep

Here we will provide a mathematical tutorial on the Wake-Sleep algorithm (Hinton et al., 1995; Dayan et al., 1995), which is one candidate biologically plausible learning

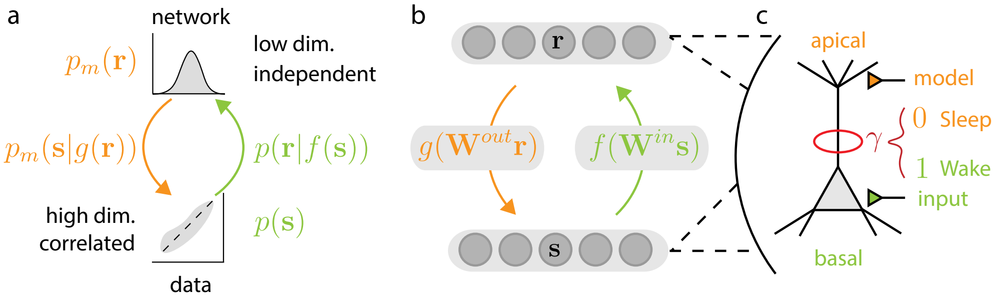


Figure S2: **The Wake-Sleep algorithm. a.** The four components of a good representation: $p_m(\mathbf{s}|\mathbf{r})$ and $p(\mathbf{r}|\mathbf{s})$ map $\mathbf{r}$ to $\mathbf{s}$ and back again from $\mathbf{s}$ to $\mathbf{r}$, respectively. $p_m(\mathbf{r})$ defines 'useful' features of a neural representation by constraining its topology. $p(\mathbf{s})$ provides the environmental input distribution, which the neural representation must match. **b.** The architecture of the Wake-Sleep algorithm: the decoder, $g(\mathbf{W}^{out}\mathbf{r})$ maps $\mathbf{r}$ to $\mathbf{s}$, and the forward map, $f(\mathbf{W}^{in}\mathbf{s})$ maps $\mathbf{s}$ to $\mathbf{r}$. **c.** Physically, these maps correspond to a multicompartmental pyramidal neuron model for each layer, where the 'model' synapses are on the apical dendrites, and the 'forward map' synapses are on the basal dendrites. $\gamma$ gates which synapses determine neural activity, putting the network in the Wake phase $\gamma = 1$ or the Sleep phase $\gamma = 0$.

algorithm for constructing a representation in sensory cortices. We will first provide one possible formulation of representation learning as an optimization problem (Roweis and Ghahramani, 1999), and then introduce the Wake-Sleep algorithm[3], showing how the components necessary to the algorithm could be mapped onto a multicompartmental dendritic neuron model with local synaptic learning. We will then discuss how the algorithm can be extended beyond our simplified introduction.

## D.1 Defining a good objective

Suppose that at any given moment in time, a neural network is receiving sensory stimuli $\mathbf{s}$ from its environment. Our first challenge is to articulate what it would mean to form a good neural representation $\mathbf{r}$ of these stimuli (Fig. S2a). First of all, 'represented' stimuli should be decodable from neural firing rates, i.e. there should exist a mapping $g(\cdot) : \mathbb{R}^{N^r} \rightarrow \mathbb{R}^{N^s}$ such that $\mathbf{s} \approx g(\mathbf{r})$. Second, we will also argue that neural firing rates should be decodable from *stimuli*, i.e. there should exist a mapping $f(\cdot) : \mathbb{R}^{N^s} \rightarrow \mathbb{R}^{N^r}$ such that $\mathbf{r} \approx f(\mathbf{s})$—this means that there cannot be 'extra' features of neural activity that are not contained within the stimuli themselves. This amounts to postulating an approximately bijective relationship between stimuli and firing rates. It means that neural activities should directly correspond to stimuli that have been received.

If these two requirements were sufficient, we might want to simply have one neuron per stimulus dimension, and have it faithfully replicate its immediate input as accu-

[3]For another excellent tutorial with more of a machine learning focus, see (Kirby, 2006).

rately as possible, i.e. we would take $f(\mathbf{s}) = \mathbb{I}\mathbf{s}$ and $g(\mathbf{r}) = \mathbb{I}\mathbf{r}$, where $\mathbb{I}$ is an identity matrix, so that $\mathbf{r} = \mathbb{I}\mathbf{s} = \mathbf{s}$. This identity transformation is obviously not useful, which makes one wonder—what does it mean for a transformation to be useful? Most, if not all unsupervised machine learning and neuroscientific conceptions of a 'useful' representation reduce to some formulation of either metabolic or coding efficiency. Approaches within this 'efficiency' umbrella include dimensionality reduction (Roweis and Ghahramani, 1999), clustering (Illing et al., 2021; Dayan et al., 1995), gain control (Simoncelli and Heeger, 1998), whitening/factorization (Rezende et al., 2014), and sparsity (Simoncelli and Olshausen, 2001). Each of these definitions of 'usefulness' can be formulated as statements about the distribution of neural activities, independent of particular received stimuli, e.g. there are fewer neurons than stimulus dimensions (dimensionality reduction), neural activations occupy roughly discrete clusters in state space (clustering), neurons tend to be uncorrelated with one another (whitening/factorization), or neurons typicaly have low, sparse firing rates (gain control/sparsity/metabolic efficiency). In our formulation, ultimately learning will be unsupervised because we have made *a priori* determinations of what constitutes an efficient representation, and seek to transform incoming data to match those determinations.

Under our definition outlined so far, there are four components of a representation: the stimuli $\mathbf{s}$ themselves, distributed according to some probability distribution $p(\mathbf{s})$ determined by the environment; a decoder, which we will formulate probabilistically as $p_m(\mathbf{s}|g(\mathbf{r};\theta_m))$, which models the probability of $\mathbf{s}$ given our mapping from neural firing rates $\mathbf{r}$; a forward mapping from $\mathbf{s}$ to $\mathbf{r}$, which we will also formulate probabilistically as $p(\mathbf{r}|f(\mathbf{s};\theta))$; and our definition of efficiency, which dictates how neural firing rates 'should' be distributed, independently of stimuli themselves $p_m(\mathbf{r})$. Notice that here we have parameterized the forward map $p(\mathbf{r}|f(\mathbf{s};\theta))$ and the decoder (inverse map) $p_m(\mathbf{s}|g(\mathbf{r};\theta_m))$: once we formulate our objective, these will be the parameters that are adjusted to minimize it. $p(\mathbf{s})$—the environmental data distribution—obviously cannot change, but we could (and in practice would often want to) parameterize $p_m(\mathbf{r})$ and also fit those parameters. We have formulated our four components using probability distributions: after describing our objective function in these terms, we will show one possible way of mapping the components onto neural architecture.

Now, we have evocatively organized our components into two groups: $p_m(\mathbf{s}|g(\mathbf{r};\theta_m))$ and $p_m(\mathbf{r})$, versus $p(\mathbf{s})$ and $p(\mathbf{r}|f(\mathbf{s};\theta))$. The first group forms a joint distribution $p_m(\mathbf{r},\mathbf{s};\theta_m)$ which has the subscript $m$ to indicate that it is a generative *model* of the data. Ideally, if its parameters were accurately fit, we could sample $\mathbf{r} \sim p_m(\mathbf{r})$, and then sample $\mathbf{s} \sim p_m(\mathbf{s}|g(\mathbf{r};\theta_m))$ and get a stimulus that looks like realistic environmental data. The second group also forms a joint distribution $p(\mathbf{r},\mathbf{s};\theta)$, which amounts to a forward mapping: we could receive a stimulus from the environment, and then have the probability distribution for firing rates $\mathbf{r}$ that correspond to it. Organizing our models in this way will allow us to achieve biophysical realism: $g(\cdot;\theta)$ and $f(\cdot;\theta)$ will correspond to actual synaptic connections in a model neural network. In practice, ordinary perception as we traditionally conceive of it would correspond to the forward mapping $f(\cdot;\theta)$. Interestingly, at the end of our derivation, it will

become clear how an additional representational feature, 'detachability' (Clark and Toribio, 1994)—a mechanism to activate neurons in the absence of the sensory stimuli that correspond to them—will be an emergent property of our formulation. We will show how a neural system might be able to leverage the $g(\cdot;\theta_m)$ to accomplish 'detachment', which one might imagine mapping perceptually to imagination, planning, prediction, hallucination, or possibly dreaming in different contexts.

For our representation to be good, the forward map should match its inverse, i.e. $p(\mathbf{r}, \mathbf{s}; \theta) \approx p_m(\mathbf{r}, \mathbf{s}; \theta_m)$. We could imagine formulating many objective functions that could accomplish this goal, but most of them will not accommodate an approximate optimization algorithm that will end up corresponding to a viable normative plasticity model. We will select the Kullback-Liebler (KL) divergence between these two distributions, precisely because it will produce such a normative plasticity model. Notice, though our presentation of the derivation is top-down, it is disingenuous to characterize normative plasticity model development strictly as top-down: locality would not magically emerge from an arbitrary choice of objective function, but rather this choice of objective function is superior to its many alternatives only *because* it produces locality (we won't be able to see why locality emerges until after we have defined $p$ and $p_m$ explicitly and have derived parameter updates). We take our objective function to be:

$$\begin{aligned}\mathcal{L}_{Wake} &= D_{KL}(p(\mathbf{r}, \mathbf{s}; \theta)||p_m(\mathbf{r}, \mathbf{s}; \theta_m)) \\ &= \int \ln\left(\frac{p(\mathbf{r}, \mathbf{s}; \theta)}{p_m(\mathbf{r}, \mathbf{s}; \theta_m)}\right) p(\mathbf{r}, \mathbf{s}; \theta) d\mathbf{r} d\mathbf{s}. \end{aligned} \tag{31}$$

We have evocatively named this loss $\mathcal{L}_{Wake}$ because we will be optimizing this objective function during the Wake phase of the algorithm. We will also later appeal to the opposite KL divergence, which we will be optimizing during the Sleep phase:

$$\begin{aligned}\mathcal{L}_{Sleep} &= D_{KL}(p_m(\mathbf{r}, \mathbf{s}; \theta_m)||p(\mathbf{r}, \mathbf{s}; \theta)) \\ &= \int \ln\left(\frac{p_m(\mathbf{r}, \mathbf{s}; \theta_m)}{p(\mathbf{r}, \mathbf{s}; \theta)}\right) p_m(\mathbf{r}, \mathbf{s}; \theta_m) d\mathbf{r} d\mathbf{s}. \end{aligned} \tag{32}$$

These objectives share a global minimum ($p_m = p$), if it exists, but are not the same objective function, because unlike a traditional distance metric, the KL divergence is not symmetric. However, *near* the global minimum, they become approximately equivalent (Dayan et al., 1995; Bredenberg et al., 2021), which will be an important consideration in assessing the convergence properties of the Wake-Sleep algorithm. Unlike REINFORCE, which will work for any reward function $R(\mathbf{r}, \mathbf{s})$, the Wake-Sleep algorithm will only work for objectives formulated in this way: in this case the choice of objective function is intimately related to the resultant plasticity rule.

### D.1.1 Equivalence to the Evidence Lower Bound*

It should be noted that $\mathcal{L}_{Wake}$ has a long history in unsupervised machine learning, and does not always appear in the context of training a sensory representational system through normative plasticity. In fact, minimizing $\mathcal{L}_{Wake}$ is equivalent to minimizing the variational free energy or maximizing the evidence lower bound (ELBO), the objective underlying the variational autoencoder (Rezende et al., 2014; Kingma and Welling, 2014) and the Expectation-Maximization algorithm for latent state models (Roweis and Ghahramani, 1999). Here, to help relate to the broader literature, we will elaborate on this equivalence for the interested reader. This section is a technical aside, which the uninterested reader may safely skip. In traditional machine learning terms, as we will see, the $\mathcal{L}_{Wake}$ objective is equivalent to maximizing the ELBO, and will fit a generative model $p_m(\mathbf{r}, \mathbf{s}; \theta_m)$ to data, as well as train a forward map $p(\mathbf{r}|\mathbf{s}; \theta)$ to perform approximate Bayesian inference with respect to that model (i.e. we want $p(\mathbf{r}|\mathbf{s}; \theta) \approx p_m(\mathbf{r}|\mathbf{s}; \theta_m)$).

To fit a generative model to data, we would typically use maximum likelihood estimation: we would find the parameters of our generative model $p_m(\mathbf{r}, \mathbf{s}; \theta_m)$ that match the distribution of data points as accurately as possible by minimizing with respect to $\theta$:

$$D_{KL}(p(\mathbf{s})||p_m(\mathbf{s}; \theta_m)) = \int \ln\left(\frac{p(\mathbf{s})}{p_m(\mathbf{s}; \theta_m)}\right) p(\mathbf{s}) d\mathbf{s}. \tag{33}$$

When this objective is 0, samples drawn from $p_m(\mathbf{s}; \theta_m)$ will be indistinguishable from samples drawn from $p(\mathbf{s})$, indicating that we have an accurate model of the data distribution. But we are not only interested in fitting a generative model: when our network receives a stimulus $\mathbf{s}$, we would like it to infer the probability distribution over latent representational states that could correspond to that stimulus, $p_m(\mathbf{r}|\mathbf{s}; \theta_m)$. However, we haven't defined this quantity, only $p_m(\mathbf{r})$ and $p_m(\mathbf{s}|\mathbf{r}; \theta_m)$. From a purely machine learning perspective, we might just try to compute $p_m(\mathbf{r}|\mathbf{s}; \theta_m)$ explicitly using Bayes' Theorem:

$$p_m(\mathbf{r}|\mathbf{s}; \theta_m) = \frac{p_m(\mathbf{r}) p_m(\mathbf{s}|\mathbf{r}; \theta)}{\int p_m(\mathbf{r}) p_m(\mathbf{s}|\mathbf{r}; \theta) d\mathbf{r} d\mathbf{s}}, \tag{34}$$

and for simple generative models this might work. However, for complex, nonlinear models, calculating the high-dimensional integral in the denominator analytically is impossible, and approximating it through Monte Carlo methods is time consuming to the point of intractability. This is motivation enough for machine learning applications, but further, it is not clear how biological system could compute such an integral rapidly upon receiving a single stimulus. So instead, we might try a different approach. We can take our explicitly defined and parameterized forward map $p(\mathbf{r}|\mathbf{s}; \theta)$ and train it to approximate $p_m(\mathbf{r}|\mathbf{s}; \theta_m)$ as closely as possible by minimizing the expected KL divergence:

$$\mathbb{E}\left[D_{KL}(p(\mathbf{r}|\mathbf{s};\theta)||p_m(\mathbf{r}|\mathbf{s};\theta_m))\right]_{p(\mathbf{s})} = \int \ln\left(\frac{p(\mathbf{r}|\mathbf{s};\theta)}{p_m(\mathbf{r}|\mathbf{s};\theta_m)}\right) p(\mathbf{r}|\mathbf{s};\theta)p(\mathbf{s})d\mathbf{r}d\mathbf{s}. \quad (35)$$

If objective is approximately 0, then we do not need to perform Bayes' theorem to calculate the posterior $p_m(\mathbf{r}|\mathbf{s};\theta_m)$, because we have access to a perfect (or near-perfect) approximation $p(\mathbf{r}|\mathbf{s};\theta)$ that we can calculate explicitly or sample from. If $p(\mathbf{r}|\mathbf{s};\theta)$ is parameterized appropriately, this is usually much easier, and potentially could be implemented by a neural network. Now we have two objectives that we want to minimize: one to fit our generative model, and the other to perform approximate inference. It seems natural to add them and minimize them jointly. First, we notice that adding our second objective defines the following inequality:

$$D_{KL}(p(\mathbf{s})||p_m(\mathbf{s};\theta_m)) \leq D_{KL}(p(\mathbf{s})||p_m(\mathbf{s};\theta_m)) + \mathbb{E}\left[D_{KL}(p(\mathbf{r}|\mathbf{s};\theta)||p_m(\mathbf{r}|\mathbf{s};\theta_m))\right]_{p(\mathbf{s})}. \quad (36)$$

due to the positivity of the KL divergence. Second, we note that adding these two objectives together really just gives us $\mathcal{L}_{Wake}$:

$$\begin{aligned} D_{KL}(p(\mathbf{s})||p_m(\mathbf{s};\theta_m)) + \mathbb{E}\left[D_{KL}(p(\mathbf{r}|\mathbf{s};\theta)||p_m(\mathbf{r}|\mathbf{s};\theta_m))\right]_{p(\mathbf{s})} &= D_{KL}(p(\mathbf{r},\mathbf{s};\theta)||p_m(\mathbf{r},\mathbf{s};\theta)) \quad (37) \\ &= \mathcal{L}_{Wake}, \quad (38) \end{aligned}$$

where the first equality follows from adding Eqs. 33 and 35 and using the properties of the logarithm and expectations.

This alternative construction demonstrates that minimizing that our objective function $\mathcal{L}_{Wake}$ trains our system to perform two separate model-fitting functions: training a generative model and training an approximate inference distribution. From here we can also see its equivalence to the variational free energy and the ELBO:

$$\begin{aligned} D_{KL}(p(\mathbf{s})||p_m(\mathbf{s};\theta_m)) &\leq \mathcal{L}_{Wake} \quad (39) \\ &= \int \ln\left(\frac{p(\mathbf{r},\mathbf{s};\theta)}{p_m(\mathbf{r},\mathbf{s};\theta_m)}\right) p(\mathbf{r},\mathbf{s};\theta)d\mathbf{r}d\mathbf{s} \quad (40) \\ &= \int \ln\left(\frac{p(\mathbf{r}|\mathbf{s};\theta)}{p_m(\mathbf{r},\mathbf{s};\theta_m)}\right) p(\mathbf{r},\mathbf{s};\theta)d\mathbf{r}d\mathbf{s} + \int (\ln p(\mathbf{s}))\, p(\mathbf{r}|\mathbf{s};\theta)p(\mathbf{s})d\mathbf{r}d\mathbf{s} \quad (41) \\ &= \int \ln\left(\frac{p(\mathbf{r}|\mathbf{s};\theta)}{p_m(\mathbf{r},\mathbf{s};\theta_m)}\right) p(\mathbf{r},\mathbf{s};\theta)d\mathbf{r}d\mathbf{s} + \int (\ln p(\mathbf{s}))\, p(\mathbf{s})d\mathbf{s}. \quad (42) \end{aligned}$$

Now, by definition $D_{KL}(p(\mathbf{s})||p_m(\mathbf{s};\theta_m)) = \int (\ln p(\mathbf{s}))\, p(\mathbf{s})d\mathbf{s} - \int (\ln p_m(\mathbf{s};\theta_m))\, p(\mathbf{s})d\mathbf{s}$, the first term of which also appears on the right hand side of our inequality. Furthermore, $\int (\ln p(\mathbf{s}))\, p(\mathbf{s})d\mathbf{s}$ is not a function $\theta_m$ or $\theta$, so from the perspective of

optimization, it is an irrelevant additive constant. We subtract it from both sides to get:

$$-\int (\ln p_m(\mathbf{s};\theta_m))\, p(\mathbf{s})d\mathbf{s} \leq \int \ln\left(\frac{p(\mathbf{r}|\mathbf{s};\theta)}{p_m(\mathbf{r},\mathbf{s};\theta_m)}\right) p(\mathbf{r},\mathbf{s};\theta)d\mathbf{r}d\mathbf{s}. \tag{43}$$

This expression on the left is the negative log-likelihood, and the expression on the right is the variational free energy, which is the negative of the ELBO. This shows that $\mathcal{L}_{Wake}$ and the variational free energy differ only by an additive constant from the perspective of optimization: minimizing one is the same as minimizing the other. Similarly, $\mathcal{L}_{Sleep}$ corresponds to an upper bound on the reverse KL divergence, $D_{KL}(p_m(\mathbf{s};\theta_m)||p(\mathbf{s}))$.

## D.2 Defining $p$ and $p_m$

Let us start by selecting three features of our representation that we think will be useful, i.e. efficient. First, we want our neurons to be metabolically efficient: a biological system cannot have neurons wasting energetic resources by firing too much (Simoncelli, 2003). One way of requiring this would be to stipulate that the squared norm of our neural firing rate vector, $||\mathbf{r}||_2^2$ lies within some reasonable range of activation values. Second, we want to reduce the dimensionality of our representation: many naturalistic datasets are low-dimensional, and it may be wasteful to represent some high-dimensional features of stimuli that are just due to sensor noise. To accomplish this, we will stipulate that $N_r \ll N_s$, where $N_r$ is the representation's dimensionality, and $N_s$ is the stimulus dimension. Third, we will require that individual neural activations should be independent from one another, which will allow individual neurons to extract important features of the data without requiring full knowledge of the activity of other neurons in the representation. To achieve a representation that embodies these three desired features, we define $p_m(\mathbf{r})$ as follows:

$$p_m(\mathbf{r}) \sim \mathcal{N}(0, 1), \tag{44}$$

i.e. we will require that the representation, averaged over stimuli, will match an $N_r$-dimensional multivariate normal distribution, where individual axes $\mathbf{r}_i$ are independent from one another (uncorrelated), and where the normal distribution naturally restricts the probable range of neural activities to lie within bounds determined by the variance (arbitrarily set to 1). Though this distribution captures several intuitions for how neural representations should function, it is clearly a toy model for several reasons: it does not restrict firing rates to be positive, it does not allow for activities to be discrete spikes, it does not account for temporal dynamics, etc. We will discuss later how each of these extensions have been done before, but for now, many features of our model $p_m(\mathbf{r},\mathbf{s})$ and our forward map $p(\mathbf{r}|\mathbf{s})$ will be unrealistic for didactic purposes.

Now we define the probabilistic decoder $p_m(\mathbf{s}|\mathbf{r})$ (Fig. S2b), which takes neural firing rates and produces estimates of stimuli, as follows:

$$p_m(\mathbf{s}|\mathbf{r};\mathbf{W}^{out}) \sim \mathcal{N}(g(\mathbf{W}^{out}\mathbf{r}), \sigma_s), \tag{45}$$

where $g(\cdot)$ is an arbitrary nonlinearity, and $\sigma_s^2$ is the variance of the decoder. In this probability distribution, we will treat the $N_s \times N_r$ matrix $\mathbf{W}^{out}$ as a free parameter which we will train to optimize our objective.

Similarly, we can define the forward map $p(\mathbf{r}|\mathbf{s})$, which takes environmental stimuli and produces firing rates, as follows:

$$p(\mathbf{r}|\mathbf{s}; \mathbf{W}^{in}) = \mathcal{N}(f(\mathbf{W}^{in}\mathbf{s}), \sigma_r), \tag{46}$$

where $f(\cdot)$ is an arbitrary (potentially different) nonlinearity, and $\sigma_r$ will ultimately correspond to intrinsic neural variability. Here, the $N_r \times N_s$ matrix $\mathbf{W}^{in}$ is the free parameter. Thus, $\mathbf{W}^{in}$ and $\mathbf{W}^{out}$, are the free parameters in our simple construction.

We have not yet made clear how these parameters and functions could map onto an actual neural architecture: we will do this after defining the learning algorithm, so that it is clear what the necessary components of the algorithm are. Interestingly, we do not have to define $p(\mathbf{s})$ at all. This distribution is determined by the environment. In fact, a learning system should ideally be as agnostic as possible to the specific form of $p(\mathbf{s})$ as possible, in order to be able to adapt strange and unforeseen changes in the statistics of the world. The Wake-Sleep algorithm is ideal in that it makes little-to-no assumption about $p(\mathbf{s})$, but as we will see, it may perform poorly if it is not possible to obtain a close match between $p$ and $p_m$. This might occur if the environmental distribution of $\mathbf{s}$ is much higher dimensional than the number of neurons, or is in some other way more complex than the generative model.

## D.3 Approximating the loss gradient

Having defined our objective function and probability distributions $p$ and $p_m$, we can now derive the Wake-Sleep algorithm. First, we will show that we can obtain a promising update for $\mathbf{W}^{out}$ by performing gradient descent on $\mathcal{L}_{Wake}$ (the Wake phase of learning). We will next show that we can obtain a similarly promising update for $\mathbf{W}^{in}$ by performing gradient descent on $\mathcal{L}_{Sleep}$ (the Sleep phase of learning). One might easily wonder why we did not perform gradient descent on $\mathcal{L}_{Wake}$ with respect to $\mathbf{W}^{in}$, instead of $\mathcal{L}_{Sleep}$: we will next show why it would be a bad idea to do this. Lastly, we will describe two perspectives on how these resultant updates can be viewed as a unified form of approximate optimization.

### D.3.1 Wake

We start by calculating the negative gradient of $\mathcal{L}_{Wake}$ with respect to a particular parameter $\mathbf{W}_{ij}^{out}$ from $p_m(\mathbf{s}|\mathbf{r}; \mathbf{W}^{out})$:

$$-\frac{d\mathcal{L}_{Wake}}{d\mathbf{W}_{ij}^{out}} = -\frac{d}{d\mathbf{W}_{ij}^{out}} \int \ln\left(\frac{p(\mathbf{r},\mathbf{s};\mathbf{W}^{in})}{p_m(\mathbf{r},\mathbf{s};\mathbf{W}^{out})}\right) p(\mathbf{r},\mathbf{s};\mathbf{W}^{in}) d\mathbf{r}d\mathbf{s} \tag{47}$$

$$= -\frac{d}{d\mathbf{W}_{ij}^{out}} \int \left[\ln p(\mathbf{r},\mathbf{s};\mathbf{W}^{in}) - \ln p_m(\mathbf{s}|\mathbf{r};\mathbf{W}^{out}) - \ln p_m(\mathbf{r})\right] p(\mathbf{r},\mathbf{s};\mathbf{W}^{in}) d\mathbf{r}d\mathbf{s} \tag{48}$$

$$= -\int \frac{d}{d\mathbf{W}_{ij}^{out}} \left[\ln p(\mathbf{r},\mathbf{s};\mathbf{W}^{in}) - \ln p_m(\mathbf{s}|\mathbf{r};\mathbf{W}^{out}) - \ln p_m(\mathbf{r})\right] p(\mathbf{r},\mathbf{s};\mathbf{W}^{in}) d\mathbf{r}d\mathbf{s} \tag{49}$$

$$= \int \left[\frac{d}{d\mathbf{W}_{ij}^{out}} \ln p_m(\mathbf{s}|\mathbf{r};\mathbf{W}^{out})\right] p(\mathbf{r},\mathbf{s};\mathbf{W}^{in}) d\mathbf{r}d\mathbf{s} \tag{50}$$

Plugging in the probability density function for $p_m(\mathbf{s}|\mathbf{r};\mathbf{W}^{out}) \sim \mathcal{N}(g(\mathbf{W}^{out}\mathbf{r}), \sigma_s^2)$, we end up with:

$$-\frac{d\mathcal{L}_{Wake}}{d\mathbf{W}_{ij}^{out}} = \int \left[\frac{d}{d\mathbf{W}_{ij}^{out}} \frac{1}{2\sigma_s^2} \sum_{i=0}^{N_s} (\mathbf{s} - g(\mathbf{W}^{out}\mathbf{r}))^2\right] p(\mathbf{r},\mathbf{s};\mathbf{W}^{in}) d\mathbf{r}d\mathbf{s}. \tag{51}$$

Similar to our derivation for REINFORCE, we see that for a particular weight $\mathbf{W}_{ij}^{out}$, $\frac{dg_l(\mathbf{W}^{out}\mathbf{r})}{d\mathbf{W}_{ij}^{out}} = 0$ if $i \neq l$. Thus, we have:

$$-\frac{d\mathcal{L}_{Wake}}{d\mathbf{W}_{ij}^{out}} = \int \frac{1}{\sigma_s^2} \left[(\mathbf{s}_i - g_i(\mathbf{W}^{out}\mathbf{r}))g_i'(\mathbf{W}^{out}\mathbf{r})\mathbf{r}_j\right] p(\mathbf{r},\mathbf{s};\mathbf{W}^{in}) d\mathbf{r}d\mathbf{s}. \tag{52}$$

Again, similar to REINFORCE, we can approximate this update as the network actively 'perceives': we receive a sampled environmental stimulus $\mathbf{s}^{(k)}$, and then sample from the probability distribution $p(\mathbf{r}|\mathbf{s}^{(k)};\mathbf{W}^{in})$ to obtain a firing rate sample $\mathbf{r}^{(k)}$. Then across $K$ samples, we calculate the approximate parameter update:

$$\Delta\mathbf{W}_{ij}^{out} \propto \frac{1}{\sigma_s^2 K} \sum_{k=0}^{K} \left[(\mathbf{s}_i^{(k)} - g_i(\mathbf{W}^{out}\mathbf{r}^{(k)}))g_i'(\mathbf{W}^{out}\mathbf{r}^{(k)})\mathbf{r}_j^{(k)}\right] \approx -\frac{d\mathcal{L}_{Wake}}{d\mathbf{W}_{ij}^{out}}. \tag{53}$$

If we want learning to be able to occur online (Section 2.5), then we can take $K = 1$, and sacrifice some precision of our estimate. This update has the form of a prediction error, where the the error between the true stimulus $\mathbf{s}_i^{(k)}$ and the network's decoded estimate $g_i(\mathbf{W}^{out}\mathbf{r}^{(k)})$ combine with presynaptic inputs $r_j^{(k)}$ to produce parameter updates. In Section D.4 we will analyze in detail how this parameter update could correspond to a local synaptic update for a particular neuron model.

### D.3.2 Sleep

So far, other than performing stochastic gradient descent over $K$ samples, we have introduced no approximation into our algorithm. We might be tempted to perform gradient descent on $\mathcal{L}_{Wake}$ with respect to $\mathbf{W}^{in}$ too: though we will defer the discussion of this point for later, it turns out to be a bad idea (see Section D.4.1). Instead, we will perform an *almost identical* procedure, but perform gradient descent on $\mathcal{L}_{Sleep}$ instead. As discussed in Section 2.1, one way of interpreting this change in loss is that we now have two different sets of parameters (i.e. synapses) in our system, $\mathbf{W}^{in}$ and $\mathbf{W}^{out}$ which are optimizing two different, albeit closely related objectives, $\mathcal{L}_{Sleep}$ and $\mathcal{L}_{Wake}$, respectively. An alternative perspective that we will discuss is that $\mathbf{W}^{in}$ is also optimizing $\mathcal{L}_{Wake}$, but is only performing an approximate gradient descent. We will discuss in Section D.4.2 how this added complexity affects the convergence and quality of the algorithm. Starting with $\mathcal{L}_{Sleep}$, we have:

$$-\frac{d\mathcal{L}_{Sleep}}{d\mathbf{W}^{in}_{ij}} = -\frac{d}{d\mathbf{W}^{in}_{ij}} \int \ln\left(\frac{p_m(\mathbf{r},\mathbf{s};\mathbf{W}^{out})}{p(\mathbf{r},\mathbf{s};\mathbf{W}^{in})}\right) p_m(\mathbf{r},\mathbf{s};\mathbf{W}^{out}) d\mathbf{r}d\mathbf{s} \tag{54}$$

$$= \int \left[\frac{d}{d\mathbf{W}^{in}_{ij}} \frac{1}{2\sigma_r^2}\sum_{i=0}^{N_r}(\mathbf{r} - f(\mathbf{W}^{in}\mathbf{s}))^2\right] p_m(\mathbf{r},\mathbf{s};\mathbf{W}^{out}) d\mathbf{r}d\mathbf{s}, \tag{55}$$

where we have followed exactly the same steps as in Eqs. 47-51.

As before, we notice that for a particular weight $\mathbf{W}^{in}_{ij}$, $\frac{df_l(\mathbf{W}^{in}\mathbf{s})}{d\mathbf{W}^{in}_{ij}} = 0$ if $i \neq l$. Thus, we have:

$$-\frac{d\mathcal{L}_{Sleep}}{d\mathbf{W}^{in}_{ij}} = \int \frac{1}{\sigma_r^2}\left[(\mathbf{r}_i - f_i(\mathbf{W}^{in}\mathbf{s}))f_i'(\mathbf{W}^{in}\mathbf{s})\mathbf{s}_j\right] p_m(\mathbf{r},\mathbf{s};\mathbf{W}^{out}) d\mathbf{r}d\mathbf{s}. \tag{56}$$

Now we can approximate this update with samples from $p_m(\mathbf{r},\mathbf{s};\mathbf{W}^{out})$. Notice that we are no longer actively perceiving via the forward mapping $p(\mathbf{r}|\mathbf{s})$ in response to sampled environmental stimuli. Instead, activity is first internally generated via $\mathbf{r}^{(k)} \sim p_m(\mathbf{r})$, before propagating to the stimulus layer to produce artificial stimuli via $\mathbf{s}^{(k)} \sim p_m(\mathbf{s}|\mathbf{r}^{(k)};\mathbf{W}^{out})$. This is termed the Sleep phase of the algorithm evocatively: an animal could not perform this type of learning while actively moving through an environment, and if it did perceive, such percepts would appear hallucinatory or dream-like, being reflective of the animal's model rather than reality. Given our $K$ samples, we calculate the approximate parameter update:

$$\Delta\mathbf{W}^{in}_{ij} \propto \frac{1}{\sigma_r^2 K}\sum_{k=0}^{K}\left[(\mathbf{r}_i^{(k)} - f_i(\mathbf{W}^{in}\mathbf{s}^{(k)}))f_i'(\mathbf{W}^{in}\mathbf{s}^{(k)})\mathbf{s}_j^{(k)}\right] \approx -\frac{d\mathcal{L}_{Sleep}}{d\mathbf{W}^{in}_{ij}}. \tag{57}$$

Now, this update should look almost equivalent to the Wake update for $\mathbf{W}^{out}$ (Eq. 53). As with the Wake update, if we want learning to occur online we can take $K = 1$. It turns out that the variability induced by this sampled approximation is *much* less than the variability induced by the REINFORCE algorithm, and is the chief reason for its superior performance and scalability (Bredenberg et al., 2021). However, it is very important to note that we are sampling from $p_m$ instead of $p$. Because our two parameter updates, Eq. 53 and Eq. 57 require sampling from two different probability distributions and individual neurons $\mathbf{r}$ could only be sampling from one probability distribution at a time, the updates are necessarily computed during different *phases*. The Wake-Sleep algorithm consists of alternating between sampling from $p$ to compute updates for $\mathbf{W}^{out}$ (the Wake phase; Eq. 53) and sampling from $p_m$ to compute updates for $\mathbf{W}^{in}$ (the Sleep phase; Eq. 57). As we discuss in Section D.4, we should be appropriately cautious about what these alternative phases could possibly mean for a biological organism.

## D.4 Assessing Wake Sleep

Having derived our Wake-Sleep parameter updates, we are finally in a position to assess the degree to which it satisfies our desiderata. We have provided a very simplified derivation of the Wake-Sleep algorithm, for a single-layer rate-based network. However, the algorithm generalizes well to recurrent, spiking, and multilayer architectures (Dayan and Hinton, 1996) (Section 2.3), and these modifications do make the algorithm more realistic as a normative plasticity model. However, it will still be very useful to show how the various components of the algorithm as we have derived it could potentially map onto realistic biological structures (Fig. S2c). First of all, we observe that both $\mathbf{s}$ and $\mathbf{r}$ need to be able to sample from either $p_m$ or $p$—for this to be possible, $\mathbf{s}$ must be *internal* to the brain, since sampling from $p_m$ affects both $\mathbf{r}$ and $\mathbf{s}$ simultaneously and would have to occur while an animal is not consciously acting in its environment. Therefore, it is best to think of $\mathbf{s}$ as a stimulus layer of neurons, and of $\mathbf{r}$ as a downstream layer of neurons receiving feedforward inputs. Next, we suppose that there is a global gating signal $\gamma$ that determines the phase of the network— if $\gamma = 1$, the network is in the Wake phase, and if $\gamma = 0$, the network is in the Sleep phase. Now we observe that the following equations will produce valid samples:

$$\mathbf{r} = \gamma f(\mathbf{W}^{in}\mathbf{s}) + (\gamma\sigma_r + (1-\gamma))\boldsymbol{\eta}_r \tag{58}$$

$$\mathbf{s} = \gamma\mathbf{s}_p + (1-\gamma)(g(\mathbf{W}^{out}\mathbf{r}) + \sigma_s\boldsymbol{\eta}_s), \tag{59}$$

where $\mathbf{s}_p \sim p(\mathbf{s})$ is an incoming sensory input, and $\boldsymbol{\eta}_s, \boldsymbol{\eta}_r \sim \mathcal{N}(0, 1)$ are sources of intrinsic noise for neurons in the stimulus, and downstream layers, respectively. Because $p_m$ and $p$ both assume exactly the same dimensionality of $\mathbf{r}$ (and $\mathbf{s}$), the only reasonable mapping of these two different sampling phases is onto one neuron with two different *modes* of activity. In Figure S2c, we show that one possible biological mapping is to propose that feedforward inputs (active when $\gamma = 1$) to the

basal dendrites of pyramidal neurons allow neurons to sample from $p$, and top-down inputs (active when $\gamma = 0$) to the apical dendrites of pyramidal neurons allows neurons to sample from $p_m$: interestingly, a corollary of this mapping is that a network could achieve 'detachability' by manipulating $\gamma$ to generate sample network states in the absence of stimuli.

It is important to note that several normative plasticity models have proposed that top-down signals to the apical dendrites could serve as some form of training signal. We will adopt a similar attitude, and now assess the locality of the Wake-Sleep parameter updates with respect to this model formulation. If we take the sample size for our updates to be $K = 1$, based on Eqs. 53 and 57, for a single pair of samples $\mathbf{r}$, $\mathbf{s}$, we have:

$$\Delta \mathbf{W}_{ij}^{in} \propto \frac{1-\gamma}{\sigma_r^2} \left[ (\mathbf{r}_i - f_i(\mathbf{W}^{in}\mathbf{s})) f_i'(\mathbf{W}^{in}\mathbf{s}) \mathbf{s}_j \right] \tag{60}$$

$$\Delta \mathbf{W}_{ij}^{out} \propto \frac{\gamma}{\sigma_s^2} \left[ (\mathbf{s}_i - g_i(\mathbf{W}^{out}\mathbf{r})) g_i'(\mathbf{W}^{out}\mathbf{r}) \mathbf{r}_j \right]. \tag{61}$$

As with REINFORCE, both $\sigma_r$ and $\sigma_s$ are proportionality constants and can be disregarded. For $\Delta \mathbf{W}_{ij}^{in}$, a basal synapse on $\mathbf{r}_i$, several variables are required. First, the same signal that gates the influence of apical versus basal inputs, $\gamma$, must also *deactivate* plasticity at basal synapses. $\gamma$ could be implemented in a neural circuit by either global inhibitory gating or by a neuromodulatory signal (Bredenberg et al., 2021)—whichever candidate signal would also have to gate plasticity. The synapse needs the postsynaptic firing rate $\mathbf{r}_i$, which is readily available, and a subtracted measure of current local to the basal compartment, $f_i(\mathbf{W}^{in}\mathbf{s})$—there is some indication that local dendritic voltage levels can affect synaptic plasticity, but the sign and exact form of this effect is variable across studies (Letzkus et al., 2006; Froemke et al., 2005; Sjöström and Häusser, 2006). As with REINFORCE, the synapse would require $f_i'(\mathbf{W}^{in}\mathbf{s})$, which is simply a monotonic function of $(\mathbf{W}^{in}\mathbf{s})_i$, and could be easily approximated; lastly, it would need the presynaptic firing rate $\mathbf{s}_j$. The information requirements for $\mathbf{W}_{ij}^{out}$ are almost exactly the same.

In terms of requiring only functions of pre- and postsynaptic activity, with the addition of some limited global context signal $\gamma$, these plasticity rules are plausibly local (Section 2.2). However, several features of this setup are unconfirmed, the most obviously testable being the Wake-Sleep sampling dynamics postulated by Eqs. 60 and 61: it seems unlikely that a neural network would entirely and synchronously switch into a 'generative' or hallucinatory regime for an extended period of time when $\gamma = 0$, and such a regime could not possibly occur in an awake, behaving animal, meaning that $\mathbf{W}^{in}$ could not be learned online (Section 2.5). However, a softer form of Wake-Sleep has been proposed (Bredenberg et al., 2021) which does allow for online learning, and does not interfere with active perception, suggesting that the principles established by Wake-Sleep may extend to more realistic formulations of $\gamma$. The strongest test (Section 2.7) of this family of algorithms is that artificially magnifying the influence of apical dendrites in a neural circuit should induce generative

sampling, i.e. hallucination; other models of apical dendritic learning (Sacramento et al., 2017; Guerguiev et al., 2017; Payeur et al., 2021; Urbanczik and Senn, 2014) do not propose this as a mechanism. Notice that this prediction requires our specific mapping of the Wake-Sleep algorithm onto neural circuitry: other interpretations are conceivable, and would have different predictions.

As we have discussed in Section D.1, the Wake-Sleep algorithm is capable of optimizing a broad range of *unsupervised* learning objectives, considerably more general than for instance Oja's rule (Oja, 1982) (though the specific toy example we provide is just a nonlinear form of probabilistic PCA). Unlike REINFORCE, the Wake-Sleep algorithm is unable to optimize reinforcement learning objectives, however, within the range of objectives that Wake-Sleep *can* optimize, it is typically much more scalable than REINFORCE (Section 2.6)[4]: in this way, it is an ideal complement, and having both algorithms or some hybridized form present in a neural circuit could be very powerful. However, the Wake-Sleep algorithm involves more approximation than REINFORCE. One could very easily wonder: since we have presented two sets of parameters in the Wake-Sleep algorithm minimizing two different objective functions, why should we expect the algorithm to converge or reliably improve performance on either objective?

To this point, we have identified two strange features of the Wake-Sleep algorithm that go hand-in-hand. First, it is strange that we should require a period of hallucinatory activity to train our parameters. Second, it is hard to interpret the convergence of an algorithm that is alternatively minimizing two slightly different objective functions: why all the work and extra conceptual baggage? Why not just do approximate gradient descent as we did with the REINFORCE algorithm and be done with it? In Section D.4.1 we will motivate why more standard gradient descent methods are not appropriate for this type of unsupervised learning, and in Section D.4.2 we will address the convergence properties of the Wake-Sleep algorithm from two different perspectives, explaining why the algorithm has such good empirical performance despite its approximations.

### D.4.1 Why gradient descent with $\mathbf{W}^{in}$ won't work

Sometimes, to genuinely understand an algorithm, it's important to understand the weaknesses of alternative approaches. For didactic reasons, we will explore what happens if we simply take the gradient of $\mathcal{L}_{Wake}$ with respect to $\mathbf{W}^{in}$. We have:

[4]Though it still performs worse than backpropagation (Kingma and Welling, 2014; Rezende et al., 2014).

$$-\frac{d\mathcal{L}_{Wake}}{d\mathbf{W}^{in}_{ij}} = -\frac{d}{d\mathbf{W}^{in}_{ij}}\int \ln\left(\frac{p(\mathbf{r},\mathbf{s};\mathbf{W}^{in})}{p_m(\mathbf{r},\mathbf{s};\mathbf{W}^{out})}\right) p(\mathbf{r},\mathbf{s};\mathbf{W}^{in})d\mathbf{r}d\mathbf{s} \tag{62}$$

$$= -\int\left[\frac{d}{d\mathbf{W}^{in}_{ij}}\ln\left(\frac{p(\mathbf{r},\mathbf{s};\mathbf{W}^{in})}{p_m(\mathbf{r},\mathbf{s};\mathbf{W}^{out})}\right)\right] p(\mathbf{r},\mathbf{s};\mathbf{W}^{in})d\mathbf{r}d\mathbf{s}$$
$$-\int \ln\left(\frac{p(\mathbf{r},\mathbf{s};\mathbf{W}^{in})}{p_m(\mathbf{r},\mathbf{s};\mathbf{W}^{out})}\right)\frac{d}{d\mathbf{W}^{in}_{ij}} p(\mathbf{r},\mathbf{s};\mathbf{W}^{in})d\mathbf{r}d\mathbf{s} \tag{63}$$

$$= -\int\left[\frac{d}{d\mathbf{W}^{in}_{ij}}\ln\left(p(\mathbf{r},\mathbf{s};\mathbf{W}^{in})\right)\right] p(\mathbf{r},\mathbf{s};\mathbf{W}^{in})d\mathbf{r}d\mathbf{s}$$
$$-\int \ln\left(\frac{p(\mathbf{r},\mathbf{s};\mathbf{W}^{in})}{p_m(\mathbf{r},\mathbf{s};\mathbf{W}^{out})}\right)\frac{d}{d\mathbf{W}^{in}_{ij}} p(\mathbf{r},\mathbf{s};\mathbf{W}^{in})d\mathbf{r}d\mathbf{s}, \tag{64}$$

where the second equality follows from the product rule, and the third equality follows from the fact that $\ln p_m(\mathbf{r},\mathbf{s};\mathbf{W}^{out})$ does not depend on $\mathbf{W}^{in}$. Interestingly, the first term in this equation is zero. To see this, we note the following sequence of identities:

$$\int\left[\frac{d}{d\mathbf{W}^{in}_{ij}}\ln\left(p(\mathbf{r},\mathbf{s};\mathbf{W}^{in})\right)\right] p(\mathbf{r},\mathbf{s};\mathbf{W}^{in})d\mathbf{r}d\mathbf{s} = \int\left[\frac{d}{d\mathbf{W}^{in}_{ij}} e^{\ln\left(p(\mathbf{r},\mathbf{s};\mathbf{W}^{in})\right)}\right] d\mathbf{r}d\mathbf{s}$$
$$= \int \frac{d}{d\mathbf{W}^{in}_{ij}} p(\mathbf{r},\mathbf{s};\mathbf{W}^{in})d\mathbf{r}d\mathbf{s}$$
$$= \frac{d}{d\mathbf{W}^{in}_{ij}}\int p(\mathbf{r},\mathbf{s};\mathbf{W}^{in})d\mathbf{r}d\mathbf{s} = \frac{d}{d\mathbf{W}^{in}_{ij}} 1 = 0. \tag{65}$$

The first term is zero, which leaves only the second term of Eq. 64. It gives us:

$$-\frac{d\mathcal{L}_{Wake}}{d\mathbf{W}^{in}_{ij}} = -\int \ln\left(\frac{p(\mathbf{r},\mathbf{s};\mathbf{W}^{in})}{p_m(\mathbf{r},\mathbf{s};\mathbf{W}^{out})}\right)\frac{d}{d\mathbf{W}^{in}_{ij}} p(\mathbf{r},\mathbf{s};\mathbf{W}^{in})d\mathbf{r}d\mathbf{s} \tag{66}$$

$$= \int \ln\left(\frac{p_m(\mathbf{r},\mathbf{s};\mathbf{W}^{out})}{p(\mathbf{r},\mathbf{s};\mathbf{W}^{in})}\right)\left(\frac{d}{d\mathbf{W}^{in}_{ij}}\ln p(\mathbf{r},\mathbf{s};\mathbf{W}^{in})\right) p(\mathbf{r},\mathbf{s};\mathbf{W}^{in})d\mathbf{r}d\mathbf{s}, \tag{67}$$

where for the second equality we have once again used the identity in Eq. 65. Fascinatingly enough, this is exactly equivalent to the REINFORCE update (Eq. 21), if we take $R(\mathbf{r},\mathbf{s}) = \ln(p/p_m)$. Though the REINFORCE update might be practical for environmental rewards that an animal might receive, this particular choice of $R(\mathbf{r},\mathbf{s})$ requires detailed knowledge of the inner workings of a neural representation.

Not only is it not possible for an environmental signal to carry this information, there is no evidence that any neuromodulatory center in the brain is able to compute such a complicated signal based on neural network activity. Thus, even though this update appears to have the form of a reward-modulated Hebbian plasticity rule, there is very little reason to believe that it is local (Section 2.2). Furthermore, this form of update is well-known to have severe scalability (Section 2.6) issues, and demonstrably performs worse than Wake-Sleep on high-dimensional datasets (Werfel et al., 2003; Bredenberg et al., 2021). The Wake-Sleep algorithm is very much a response to these failings, using a local error signal specific to each neuron, rather than correlating each neuron's activity with a global reward signal. However, the Wake-Sleep algorithm employs more approximations than REINFORCE. In Section D.4.2, we will analyze the convergence properties of Wake-Sleep.

### D.4.2 The convergence of Wake-Sleep

Currently, we have two updates that are approximating gradient descent on two different objectives: $\Delta \mathbf{W}^{out}_{ij} \approx -\lambda \frac{d\mathcal{L}_{Wake}}{d\mathbf{W}^{out}_{ij}}$, and $\Delta \mathbf{W}^{in}_{ij} \approx -\lambda \frac{d\mathcal{L}_{Sleep}}{d\mathbf{W}^{in}_{ij}}$, where $\lambda$ is a small positive learning rate. In Section 2.1, we stressed the importance of viewing plasticity updates as decreasing a *unified* objective, but here we have two. How do we know that $\Delta \mathbf{W}^{in}_{ij}$ won't *increase* $\mathcal{L}_{Wake}$ and vice versa? Clearly, $\mathcal{L}_{Sleep}$ and $\mathcal{L}_{Wake}$ are closely related: one way of resolving this difficulty is by demonstrating that $\Delta \mathbf{W}^{in}_{ij} \approx -\lambda \frac{d\mathcal{L}_{Wake}}{d\mathbf{W}^{in}_{ij}}$. In this case, during the Wake phase, the system would optimize $\mathcal{L}_{Wake}$ with respect to $\mathbf{W}^{out}$, and during the Sleep phase, it would approximately optimize the same objective with respect to $\mathbf{W}^{in}$—this would amount to an approximation of coordinate descent. In fact, under certain conditions, it turns out that this is exactly what the Wake-Sleep algorithm is doing.

To see this, we begin with the REINFORCE-like update (Eq. 67) for gradient descent on $\mathcal{L}_{Wake}$:

$$-\frac{d\mathcal{L}_{Wake}}{d\mathbf{W}^{in}_{ij}} = \int \ln\left(\frac{p_m(\mathbf{r},\mathbf{s};\mathbf{W}^{out})}{p(\mathbf{r},\mathbf{s};\mathbf{W}^{in})}\right)\left(\frac{d}{d\mathbf{W}^{in}_{ij}} \ln p(\mathbf{r},\mathbf{s};\mathbf{W}^{in})\right) p(\mathbf{r},\mathbf{s};\mathbf{W}^{in}) d\mathbf{r} d\mathbf{s}. \tag{68}$$

Interestingly, we notice that if $p_m \approx p$, then by first-order Taylor expansion, $\ln(p_m/p) \approx p_m/p - 1$. Plugging this approximation in (see (Bredenberg et al., 2021) for a more detailed justification of this approximation), we get:

$$-\frac{d\mathcal{L}_{Wake}}{d\mathbf{W}^{in}_{ij}} \approx \int \left( \frac{p_m(\mathbf{r}, \mathbf{s}; \mathbf{W}^{out})}{p(\mathbf{r}, \mathbf{s}; \mathbf{W}^{in})} - 1 \right) \left( \frac{d}{d\mathbf{W}^{in}_{ij}} \ln p(\mathbf{r}, \mathbf{s}; \mathbf{W}^{in}) \right) p(\mathbf{r}, \mathbf{s}; \mathbf{W}^{in}) d\mathbf{r} d\mathbf{s} \tag{69}$$

$$= \int \left( \frac{d}{d\mathbf{W}^{in}_{ij}} \ln p(\mathbf{r}, \mathbf{s}; \mathbf{W}^{in}) \right) p_m(\mathbf{r}, \mathbf{s}; \mathbf{W}^{out}) d\mathbf{r} d\mathbf{s} \tag{70}$$

$$= -\frac{d\mathcal{L}_{Sleep}}{d\mathbf{W}^{in}_{ij}}, \tag{71}$$

where for the first equality we have once again used the identity Eq. 65. Essentially, if a global optimum such that $p_m = p$ exists, it is shared by both $\mathcal{L}_{Wake}$ and $\mathcal{L}_{Sleep}$. Thus, we can expect the gradients of these two objective functions to behave very similarly if $p_m$ is close to $p$. Because the Wake phase (updating $\mathbf{W}^{out}$) occurs without approximation, the algorithm has the opportunity to enter this regime before the approximating Sleep phase ever occurs.

An alternative analysis of the Wake-Sleep algorithm (Dayan et al., 1995) observes that for fixed $\mathbf{W}^{out}$, $\mathcal{L}_{Sleep}$ and $\mathcal{L}_{Wake}$ share a global minimum with respect to $\mathbf{W}^{in}$ when $p_m(\mathbf{r}|\mathbf{s}; \mathbf{W}^{out}) = p(\mathbf{r}|\mathbf{s}; \mathbf{W}^{in})$, as long as there exists a $\mathbf{W}^{in}_{opt}$ such that this equality holds. If $\mathcal{L}_{Sleep}$ is convex and this global minimum is attainable, fully optimizing $\mathcal{L}_{Sleep}$ with respect to $\mathbf{W}^{in}$ during the Sleep phase is therefore guaranteed to also optimize $\mathcal{L}_{Wake}$. Therefore, as long as these to conditions of convexity and attainability of the global minimum are satisfied (they are not in general, but do hold for simple examples like Factor Analysis (Amari and Nakahara, 1999)), both phases decrease $\mathcal{L}_{Wake}$. Rather than an approximation of coordinate descent, this can be viewed as an approximation of the Expectation-Maximization (EM) algorithm (Dempster et al., 1977).

We see that there are two different ways of interpreting Wake-Sleep: first, it is an approximation of coordinate descent that becomes a better approximation the closer to the optimum it becomes. Second, under restricted conditions, Wake-Sleep can be viewed as an approximation of the EM algorithm. Both of these perspectives are conditional on assumptions about the probability models being trained, requiring a generative model $p_m(\mathbf{r}, \mathbf{s})$ and a forward map $p(\mathbf{r}|\mathbf{s})$ capable of mutually reaching good performance for an environmental stimulus distribution $p(\mathbf{s})$. Though Wake-Sleep empirically performs quite well under a variety of stimulus conditions and network models (Dayan and Hinton, 1996), these are important caveats: the comparative weakness of the demonstrations of Wake-Sleep's convergence relative to gradient descent or EM is a common point of criticism of the algorithm (Rezende et al., 2014; Kingma and Welling, 2014; Mnih and Gregor, 2014).
